# An advanced systems biology framework of feature engineering for cold tolerance genes discovery from integrated omics and non-omics data in soybean

**DOI:** 10.3389/fpls.2022.1019709

**Published:** 2022-09-30

**Authors:** Pei-Hsiu Kao, Supaporn Baiya, Zheng-Yuan Lai, Chih-Min Huang, Li-Hsin Jhan, Chian-Jiun Lin, Ya-Syuan Lai, Chung-Feng Kao

**Affiliations:** ^1^ Department of Agronomy, College of Agriculture and Natural Resources, National Chung Hsing University, Taichung, Taiwan; ^2^ Department of Resource and Environment, Faculty of Science at Sriracha, Kasetsart University, Sriracha, Thailand; ^3^ Advanced Plant Biotechnology Center, National Chung Hsing University, Taichung, Taiwan

**Keywords:** soybean, cold tolerance, feature engineering, omics and non-omics data integration, systems biology, non-parameter random forest prioritization, pathway-network analysis, sample classification

## Abstract

Soybean is sensitive to low temperatures during the crop growing season. An urgent demand for breeding cold-tolerant cultivars to alleviate the production loss is apparent to cope with this scenario. Cold-tolerant trait is a complex and quantitative trait controlled by multiple genes, environmental factors, and their interaction. In this study, we proposed an advanced systems biology framework of feature engineering for the discovery of cold tolerance genes (CTgenes) from integrated omics and non-omics (OnO) data in soybean. An integrative pipeline was introduced for feature selection and feature extraction from different layers in the integrated OnO data using data ensemble methods and the non-parameter random forest prioritization to minimize uncertainties and false positives for accuracy improvement of results. In total, 44, 143, and 45 CTgenes were identified in short-, mid-, and long-term cold treatment, respectively, from the corresponding gene-pool. These CTgenes outperformed the remaining genes, the random genes, and the other candidate genes identified by other approaches in an independent RNA-seq database. Furthermore, we applied pathway enrichment and crosstalk network analyses to uncover relevant physiological pathways with the discovery of underlying cold tolerance in hormone- and defense-related modules. Our CTgenes were validated by using 55 SNP genotype data of 56 soybean samples in cold tolerance experiments. This suggests that the CTgenes identified from our proposed systematic framework can effectively distinguish cold-resistant and cold-sensitive lines. It is an important advancement in the soybean cold-stress response. The proposed pipelines provide an alternative solution to biomarker discovery, module discovery, and sample classification underlying a particular trait in plants in a robust and efficient way.

## Introduction

Soybean [*Glycine max* (L.) Merr.] is served as one of the most economically valuable crops globally and currently is the first and fourth largest grain or oilseed crop in the world in terms of harvested area and yield, respectively (FAOSTAT, http://www.fao.org/faostat/en/#compare). Soybean is not only a dietary staple in human society, but also the material of many kinds of the processed products (e.g., soymilk, tofu, miso, and so on) and has a positive effect on the human body (Messina, 1997; Van Ee, 2009; Qin et al., 2022). In recent years, the soybean’s growing environment has faced more severe pressure, due to the extreme temperature incurred by rapid climate change (Gonçalves et al., 2021). Soybean is regarded as a cold-sensitive crop species (Robison et al., 2017). Hence, it has an urgent need to identify candidate tolerance genes for cold-stress in soybean and breed the cold-resistant cultivars to cope with its endangered growing environment.

Recently, agriculture around the world has faced more serious abiotic stresses (such as extreme temperatures, drought, flooding, and salinity), resulting in approximately 51-82 percent loss in crop yield annually (Oshunsanya et al., 2019). Therefore, efforts to enhance plants’ tolerance toward abiotic environmental stresses, such as temperature extremes, remain challenging. Cold stress is an abiotic stress factor that suppresses crop productivity, which can be divided into chilling (0~15 °C) and freezing (< 0 °C) stress (Ding et al., 2019). Both stresses can influence plants’ photosynthesis, cellular metabolism, and the production of abscisic acid (ABA) and jasmonic acid (JA), causing physiological damage during exposure. Growing tropical crops, such as soybean, rice, and corn in temperate climates (e.g., North America, North-Eastern China, Brazil.), tends to induce their cold sensitivity when exposed to chilling stress (Bandara et al., 2021). The appropriate temperature for soybean growth during the vegetative stage is 15~22 °C (Liu et al., 2008). Under chilling stress, soybean seedlings may result in growth retardation (below 15 °C), a low rate of germination, and declining vitality (below 10 °C). The decreases in the germination rate and the seedlings’ vitality happen at temperatures below 10 °C. Furthermore, temperatures below 6°C will cause little growth in soybean seedlings, severely blocking the physiological features. (Bandara et al., 2021). Cold stress imposes a significantly adverse impact on shoot height and shoot dry matter accumulation of soybean, and also declines the development of new leaves. In addition, low temperatures at flowering deter soybean’s floral initiation. Cold stress can negatively interfere with growth and development at all phenological stages in soybean and, therefore, is an enormous impediment to crop growth (Sanghera et al., 2011).

As highlighted above, there is a solid need to breed cold-tolerant soybean cultivars under climate change conditions. Cold-tolerant soybean species can resist low temperature environments to prevent chilling injury signs, such as chlorosis, necrosis, or growth retardation (Sanghera et al., 2011). Cold inducible genes in plants involve many metabolic pathways that drive the plant metabolism to respond to low temperature environment (Sanghera et al., 2011). The C-repeat/DRE binding factors (CBF/DREBs) play a vital role in cold tolerant plants. The research shows that both cold treatments, 1hr and 24hr, changed the CBF/DREBs genes transcript level significantly (adjusted *p-*value< 0.001) (Yamasaki and Randall, 2016).

Over the last decade, candidate-gene approaches based on knowledge of potential functions and physiological responses are most commonly used to search for functional or adaptively relevant loci that play key roles in a phenotypic trait of interest using a variety of experimental designs and genetic approaches, including linkage mapping (Jiang et al., 2009; Qiu et al., 2011; Zhang et al., 2012), and gene expression profiling (Yamasaki and Randall, 2016; Robison et al., 2019). Advances in high-throughput experimental technologies have dramatically generated and accumulated massive omics data and complex bioinformatics, providing the opportunity to merge new dimensions in crop improvement programs. Omics technologies such as genomics, transcriptomics, proteomics, and metabolomics, either genome wide or targeted, use a systems biology approach to characterize and quantify pools of biological molecules for comprehensively understanding the structures, functions, and their dynamics of a cell, tissue, or organism (Vailati-Riboni et al., 2017). Systems biology integrated multi-omics data across a wide range of fields (biology, informatics, data science, statistics, and computational science) involved, from different experimental backgrounds, providing a more powerful foundation, more comprehensive understanding and more meaningful insight into stress tolerance, physiology mechanism, genetic processes and others in different crop species (Pazhamala et al., 2021). Von Bertalanffy (1973) introduced the concept of systems biology and then used it to systematically integrate multi-omics data on potatoes (Acharjee et al., 2016). With the development of integration methods, it is becoming available to incorporate complex biological information from several omics and non-omics (OnO) data. Recently, Lai et al. (2021) proposed a framework of multi-dimensional databases integration, combining genomic and genotypic data, and a step function-based weighting scheme to select flooding tolerance genes. Integrating knowledge derived from genetic information and multiple omics data, coupled with bioinformatics and bioanalytical approaches, can improve gene discovery to better understand complex networks of interactions between genes, proteins, metabolites and environmental factors within a complex phenotypic trait (e.g., cold tolerance and response to cold stress).

Most abiotic stress-related traits result from the interaction of several phenotypic features with multi-environment conditions, which are complex in nature. Understanding these complex mechanisms underlying biological processes and molecular functions requires complete and precise data to characterize such features and conditions in detail. However, the integration of OnO data is not an easy task due to data complexity, data heterogeneity (e.g., different data types and formats from varying designs/technologies), data harmonization (e.g., different data scaling, normalization, standardization, and transformation), and identifiers mapping (e.g., matching gene/pathway annotations with transcripts/proteins/metabolites). Alternatively, incorporating high-dimensional omics data and low-dimensional non-omics data is still a challenge in reducing potential bias, noise, and interaction between multi OnO data. Therefore, many modelling approaches, including independent modelling, conditional modelling, and joint modelling were developed to overcome the challenges mentioned above during OnO data integration (López De Maturana et al., 2019).

Dimension reduction is an important step in the modeling process. Feature engineering is one of the effective ways to reduce the complexity of data, remove irrelevant variables, and increase the modeling efficiency (Khalid et al., 2014). Appropriate feature engineering plays an important factor in successful modeling, and the techniques of feature engineering differ from field to field (Verdonck et al., 2021). An example of application of feature engineering in the agricultural fields was applied to yield prediction, using the manpower-based (agricultural experts), the algorithm-based (random forest variable importance), and the mathematical model-based (Pearson correlation) feature selection and feature extraction to eliminate the redundant variables (Shahhosseini et al., 2020). Therefore, benefits from the feature engineering enable us to know more clearly about the interaction between environmental factor and crop yields.

The concept of gene prioritization is not new. Several prioritization approaches were proposed for complex traits in human diseases. Recently, the idea of gene prioritization was applied to rice bacterial leaf blight (Xia et al., 2013), *Arabidopsis thaliana* flowering-time (Zhai et al., 2016), and soybean flooding tolerance (Lai et al., 2021). Nevertheless, there is still some space left for improvement. The gene prioritization approaches used in these studies were evidence-based (e.g., rank, impact factor, and term frequency-based), which were highly dependent upon a specific set of features. The key challenge in gene prioritization is to precisely prioritizing a list of candidate genes accordingly and selecting important genes for a specific phenotype of interest. The random forest (RF) has been one of the most widely-known algorithms in the scientific area. The RF algorithm undergoes two random stages, bootstrapping and random feature selection. Additionally, the RF chooses features randomly to generalize over the data to prevent overfitting and provide stable generalization errors. The RF is always a better way for researchers to solve multi-class problems and cope with a large amount of data (Breiman, 2001). In this study, we employed the non-parameter random forest (NPRF) algorithm to prioritize a list of genes in a large-scale dataset to avoid false-positive results and provide a much more accurate decision during the gene prioritization stage.

A gigantic amount of biological data has been generated due to the progress of computational technologies and biological techniques, providing opportunities to identify the underlying biological phenomena through pathway analysis and crosstalk networks. The concept of pathway crosstalk describes the correlations or relationships among pathways in terms of the degree of the overlapping or sharing genes due to closely related functions. Studying pathway crosstalk at the network level in plants can not only reveal the whole architecture of the mechanism underlying a certain phenotype of interest but also can validate hypotheses experimentally about the crosstalk. Several applications of crosstalk in plants have uncovered novel findings and new insights, including hormone crosstalk on the regulation of plant defense (Aerts et al., 2021). The first application of crosstalk in soybean was introduced by Song et al. (2019) to uncover the role of BZR1-like proteins (Gm*BZLs*) in brassinosteroids signaling regulation, involved with several plant hormones and abiotic stress. Investigation of such interaction is imperative to unveil the potential crosstalk and provides a more comprehensive insight into physiological mechanisms.

Because of the increasing frequency and intensity of cold extremes and being a cold-sensitivity crop, there is an urgent need to identify the candidate genes relevant to cold-tolerant responses in soybean for breeding cold-resistant cultivars to alleviate the production loss. In particular, soybean cold-tolerant responses are complex quantitative traits controlled by many genes, environmental factors, and their integration. Therefore, we developed a systematic and comprehensive framework of feature engineering in the present study, including integrated feature selection and feature extraction for OnO data integration and genes prioritization, to identify key genes favoring cold tolerance (denoted as CTgenes) in soybeans. Here, we defined the CTgenes as significantly associated with cold tolerance or cold responses contributing to cold-related traits during the vegetative growth phase in experiments with treatments at low temperature (below 15 °C). According to the period of time of cold tolerance, we classified them into three periods, including short-term (shorter than 12hr), mid-term (between 12hr to 48 hr), and long-term (longer than 48hr) (Hannah et al., 2005). We first employed the data-ensemble methods to systematically grab and integrate valid information from collected OnO data to access valuable insights into biological events and processes. Possible sources of potential biases (i.e. selection bias and ascertainment bias) and noise were minimized during the data-ensemble stage. A scoring system was set to evaluate the varying magnitude of associations for each of the collected genes related to the cold tolerance or response to cold stress in soybean. A NPRF prioritization algorithm and statistical testing approaches were proposed to select prioritized CTgenes for short-, mid-, and long-term cold tolerance. We compared our results to other existing methods to evaluate the robustness and effectiveness of the prioritized CTgenes in a large-scale RNA-seq gene expression data (Yamasaki and Randall, 2016) under cold treatment in soybean leaves. Furthermore, a model-based pathway enrichment analysis and pathway crosstalk network were performed to gain insight into the biology of functional contexts of the CTgenes. Finally, we validated our CTgenes using genotypes data of 56 soybean samples in cold tolerance experiments. Significant contributions to scientific research may include multiple aspects through our developed systems biology pipelines. (1) Fast-precise biomarker discovery and sample classification: important key genes underlying a particular phenotype can be efficiently selected to distinguish diverse sample features. (2) Higher accuracy and fewer false-positive results: rigorous data quality control can typically produce high-quality results without information loss. (3) Cost reduction: through the pipelines, several costs in time, funding, manpower, and workforce can be minimized. (4) Application and generalization: our proposed systems biology framework can be applied to other important phenotypes (e.g. drought tolerance) in soybeans and generated for other plant species (e.g. rice) in an efficient way.

## Materials and methods

**Figure 1:**
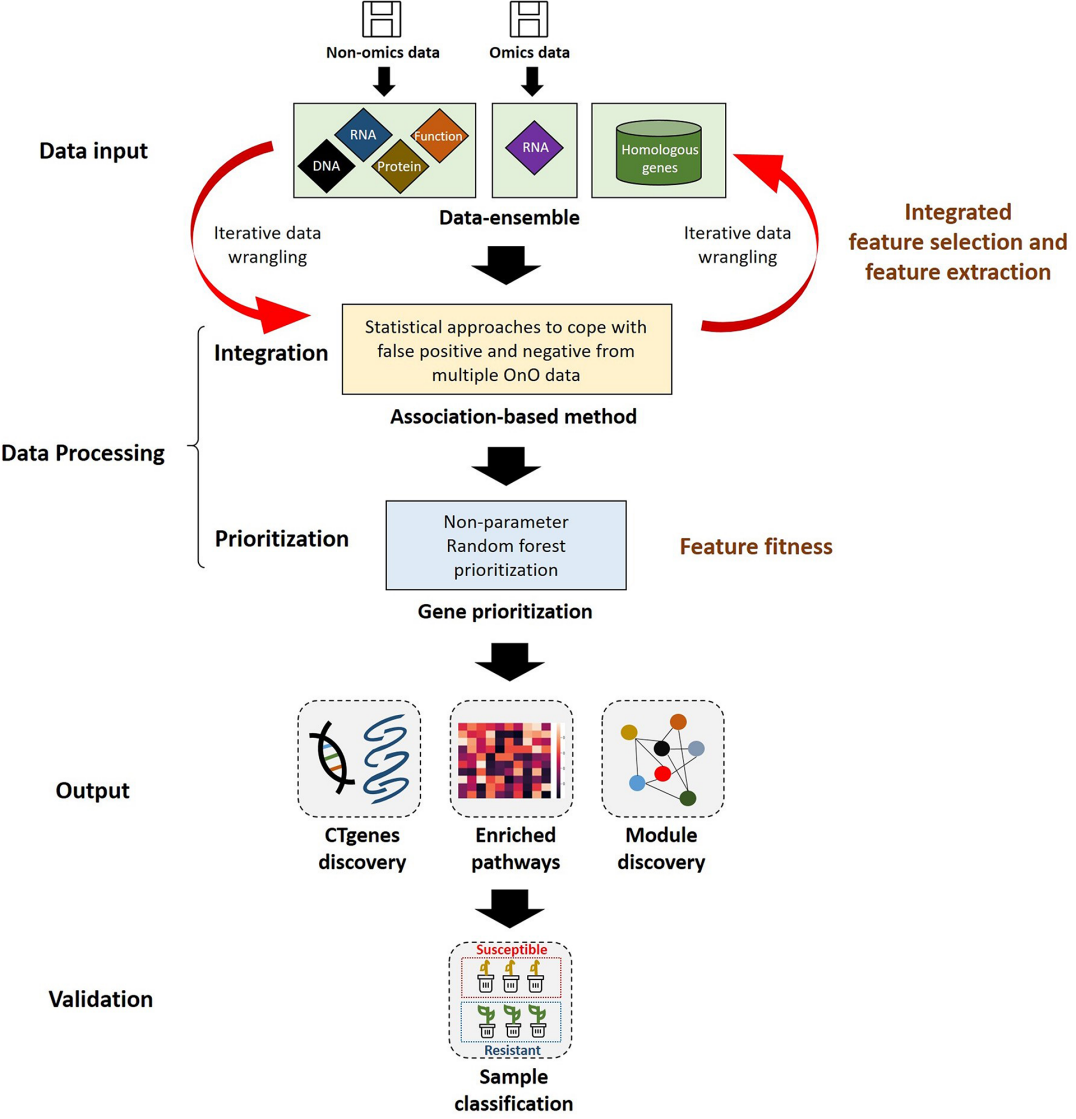
The pipeline of feature engineering for omics and non-omics (OnO) data analytic strategy. The OnO data were classified into different layers (DNA, RNA, protein, function, homologs) of information, which were regarded as the input data. Integrated feature selection and feature extraction include the data-ensemble step and the integration step (using the association-based method), which were applied to remove unwanted data (i.e., uncertainties, irrelevant and redundant data, noise, errors, and false positives) and integrate the OnO data. The data processing step consists of the OnO data integration and gene prioritization. In the feature fitness, we proposed the non-parameter random forest algorithm for gene prioritization. The steps of data-ensemble and association-based integration approach are an iterative procedure for data updates (e.g. noise or errors removal, new added data). Data output step was illustrated by the discovery of CTgenes, enriched pathways, and module discovery. Validation study step was executed by cold tolerance experiments using 55 SNP genotypes data of 56 soybean samples.

We proposed a comprehensive and systematic framework of feature engineering for OnO data integration from multiple data sources, and prioritized them for the identification of cold tolerance genes in soybean. Our framework is comprised of four steps. The first step is data input, including OnO data mining and data ensemble. The second step is data processing consisting of integration analysis and gene prioritization. Integrated feature selection and feature extraction included unwanted data (i.e., uncertainties, irrelevant and redundant data, noise, errors, and false positives) exclusion using the data-ensemble method and the OnO data integration using the association-based method in the integration analysis. Feature fitness utilized the NPRF prioritization on decision trees construction. The third step is data output comprising CTgenes discovery, pathway enrichment analysis, and crosstalk network (i.e. module discovery). The fourth step is the validation study including cold tolerance experiments and cluster analysis (i.e. sample classification). A detailed pipeline of feature engineering for the OnO data integration and analytic strategy in this study is illustrated in **Figure 1**.

### OnO data mining and wrangling

The OnO data was mined from publications and open databases available in NCBI PubMed and Google Scholar. Only data related to soybean cold tolerance or response to cold stress (temperature below 15 °C) were collected by searching keywords. The related search terms were combinations of crop and trait. Keywords for crop included ‘soybean’ and ‘*Glycine max*’. Keywords relevant to cold tolerant trait were ‘cold’, ‘freezing’, ‘cool’, ‘chilling’, ‘low temperature’, ‘hypothermia’, and ‘microtherm’. To maximize the completeness of the datasets, we collected omics data (genomics, transcriptomics, proteomics, and metabolomics) from different layers (DNA, RNA, protein, function) of information. Similarly, physiological, pathological, phenotypic, demographic and ecological data from individual studies and large-scale non-omics data were collected. We recorded different types of OnO features analyzed by different approaches in each layer. For example, genomic data was collected from genome-wide association study (GWAS), association mapping, linkage mapping, and pathway analysis at the DNA layer. Transcriptomic data were collected from gene expression, non-coding RNA, and pathway regulation in the RNA layer. Proteomic data were collected from protein-protein interaction networks (PPIN) and proteome studies in the protein layer. Metabolome data were collected from functional networks and pathway regulation studies at the function layer. Besides, model plants (*Arabidopsis thaliana* and *Medicago truncatula*) have been studied for a comprehensive understanding of soybean functional genes. Hence, they were included in the OnO data integration as a layer.

We defined inclusion criteria as soybean and cold tolerance or response to cold stress only. The exclusion criteria considered studies that were related to genetically modified studies, human and animal experiments, non-soybean traits, and other irrelevant studies. To avoid possible noise and false positive results, we only considered QTLs and targeted traits (cold tolerance or response to cold stress) were associated within a 5 centimorgan (cM) interval. Both inclusion and exclusion criteria were used to minimize potential noise and biases.

### Integrated feature selection and feature extraction

The integrated feature selection and feature extraction consists of the data-ensemble method and the OnO data integration analysis. The data-ensemble method involves several processes of data management and quality control, including data cleaning, data harmonization, data heterogeneity, and data mapping. In data cleaning process, we discarded unwanted data (i.e. duplicate, irrelevant, and not applicable data to this study) and also corrected inaccurate data (i.e. typos and incorrect data) to ensure correct and consistent data. In data harmonization process, we unified diverse data in formats, types, levels, and dimensions that were curated from different data sources into an aligned entire to ensure that data are comparable. An association-based scoring system was developed to integrate OnO data. Each marker, including single nucleotide polymorphism (SNP), simple sequence repeat (SSR), and QTL, was assigned a score to evaluate the magnitude in relation to cold tolerance or cold response in soybean. Scores for *p*-values, fold-change (FC), the logarithm of odds (LOD), degrees, and cluster coefficients were correspondingly transformed by using 10-based logarithms, absolute values, floor function, and step function to extract a suitable representation of data. Because OnO data were generated by different technologies, all scores were constrained within a reasonable range to avoid overestimated or underestimated scores extracted from a single platform in the data heterogeneity process. A detailed scoring scheme for diverse data formats across different layers is described in **Supplementary Table 1**. In the data mapping process, annotations of OnO entities (transcripts, proteins, SNPs, SSRs, and QTLs) with bioinformatics were matched to annotated genes. A window spanning 20 kb upstream/downstream of a gene was used in gene annotation mapping (Lai et al., 2021). In addition, annotated pathways (e.g., GeneOntology, GO-terms) were matched with corresponding gene sets. Finally, gene version correspondence analysis was performed to match different gene versions (Glyma v1.0, Glyma v1.1, and Glyma v2.0) (Grant et al., 2010; Schmutz et al., 2010), and unify them into Glyma v2.0 (Wm82.a2.v1) gene version (https://phytozome-next.jgi.doe.gov/info/Gmax_Wm82_a2_v1; https://soybase.org/dlpages/#annot).

**Figure 2:**
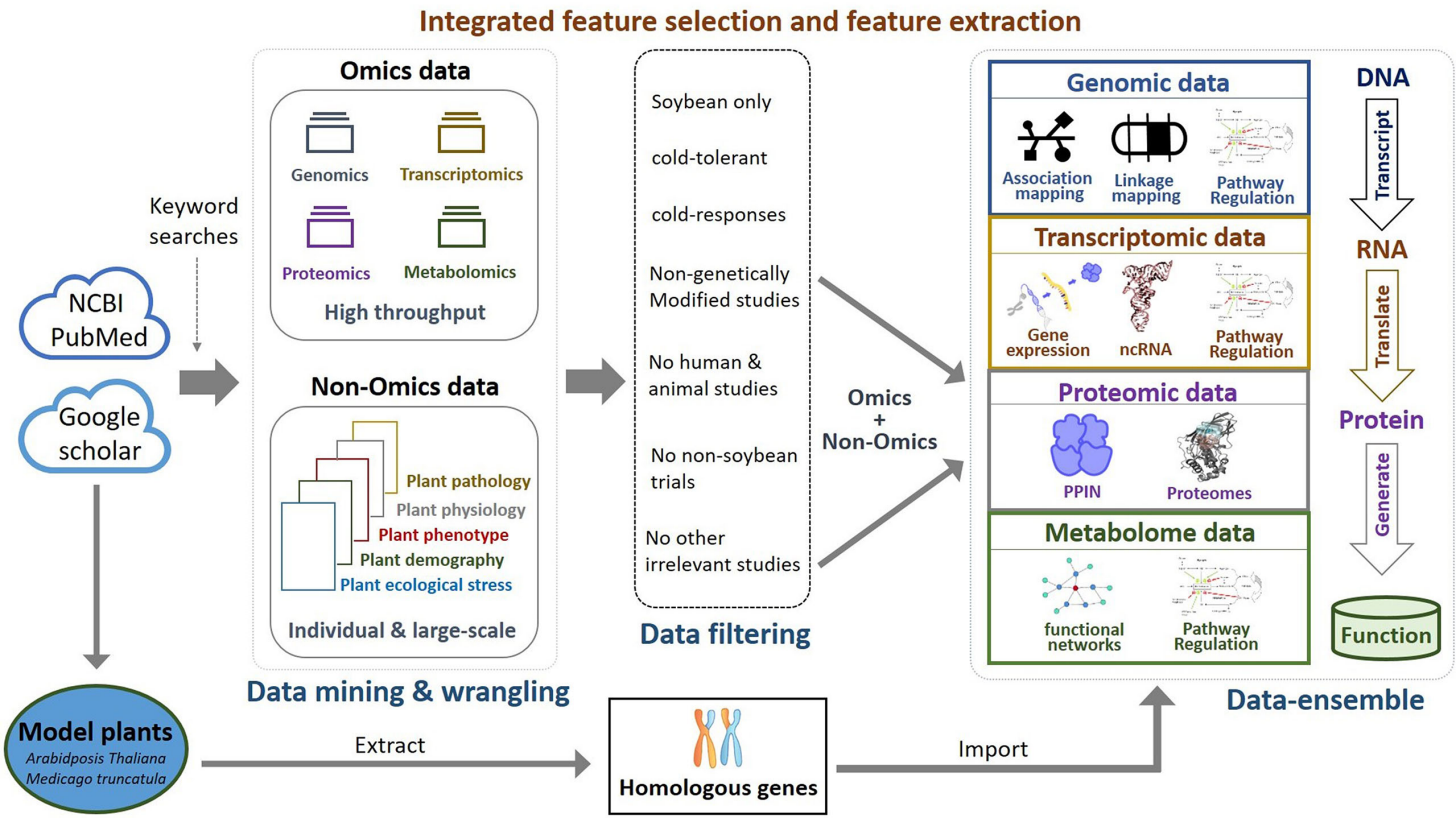
Framework of integrated feature selection and feature extraction for OnO data integration. Multiple OnO data (genomic, transcriptomic, proteomic, and metabolomic data) were classified into different information layers (DNA, RNA, protein, function, and homologs). Each layer has different types of the OnO features analyzed by different methods.

**Figure 3:**
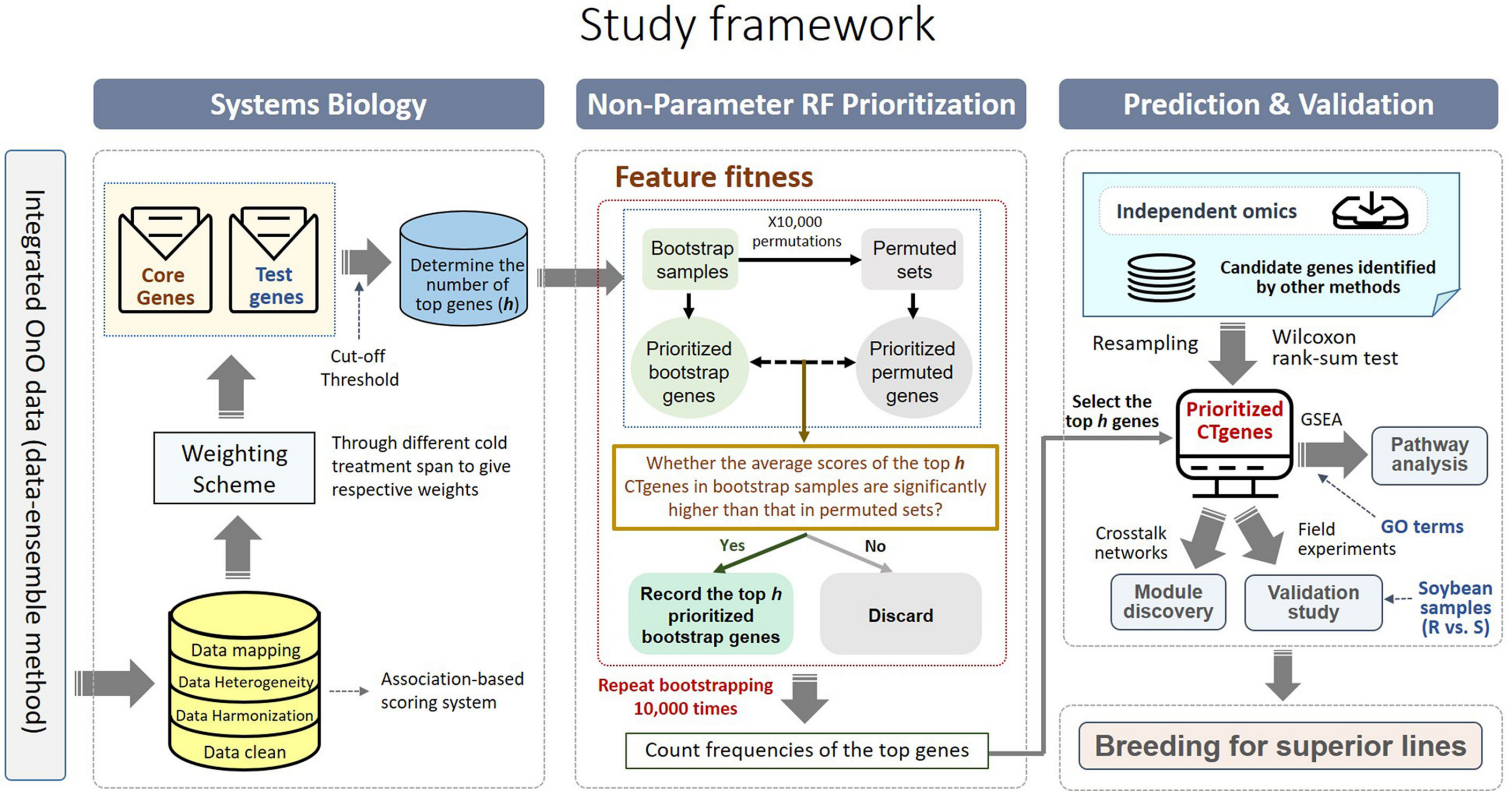
An overview of the integrated OnO data and breeding for superior lines in soybean. There are four primary steps: systems biology, non-parameter random forest prioritization, prediction and validation (pathway enrichment, module discovery, and validation study). GSEA represents gene-set enrichment analysis; GO represents gene ontology; R represents resistant varieties; S represents susceptible varieties.

Comparative genome mapping to different plant species using homologous genes with similar functions provides great potential for biomarker discovery (Gale and Devos, 1998). To extend the OnO data dimension, we included homologous genes from *Arabidopsis thaliana* and *Medicago truncatula* as the model plants. The keyword search technology was used to excavate relevant publications on cold tolerance in NCBI PubMed. Only data reported to be significantly tolerant to cold stress and validated in cold stress experiments were collected. We used BLASTP (Camacho et al., 2009) to conduct a sequence similarity search for protein sequences of the *Glycine max* genome by using alignment to match those from *Arabidopsis* and *Medicago*. Homologous proteins or genes, corresponding to soybean genes, were identified by the highest similarity in sequence searches. The similarity selection criteria included E-value less than 1.0×10^-10^ and an identity greater than 35 percent. We applied a step-function proportional to the number of references to score the importance of homologous genes in relation to cold tolerance or response to cold stress. **Figures 2**, **3** (only the ‘Systems Biology’ panel) demonstrates a novel framework of OnO data integration based on comprehensive data management and quality control rigorously and systematically. More attentively, **Figure 2** displays the framework of integrated feature selection and feature extraction for OnO data integration. The OnO data mining was implemented on keyword searches. Multiple OnO data, consisting of omics data (genomic, transcriptomic, proteomic, and metabolomic data) and non-omics data, were classified into different layers (DNA, RNA, protein, function, and homologs) with different types of features analyzed by different methods. The collected data were filtered to exclude irrelevant data from this study, and classified into DNA, RNA, protein, and function layer in the data-ensemble step. In addition, homologous genes from model plants were extracted and included in the homologs layer. The ‘Systems Biology’ panel in **Figure 3** demonstrates an overview of the integrated OnO data at the systems level. The OnO data integration was implemented based on the association-based scoring system. Feature selection and feature extraction were performed using the data-ensemble methods during data clean, data harmonization, data heterogeneity, and data mapping steps. A weighting scheme was applied through different cold treatment span to give respective weights. The test genes were compared to the core genes to determine the number of the top genes by an optimal cut-off threshold of combined score.

We defined *X^i^* (*i*=1,2,⋯,*k* ) to be the OnO matrix, with *m* layers (e.g., DNA, RNA, protein, function, and homologs) and *n_i_* features (e.g., SNPs, QTLs, mRNA, miRNA, gene, etc.). We jointly merged the *k* OnO matrices into a (*n*×*m*) OnO-specific matrix, where *n* sums over all features across all OnO matrices (i.e. $n=\sum_{i=1}^{k}n_{i})$ . For each gene, a score (denoted as $S_{j}^{l}$ , *j*=1,⋯,*n* and *l*=1,⋯*m*) was calculated for each layer using the scoring scheme mentioned above. We defined *W*_*j*_ (*j*=1,⋯,*n* ) as a weight based on the duration of cold tolerance to evaluate the capacity of exposure to cold stress for each gene. Overall, a pool of genes (denoted as the test genes) extracted from the OnO data, each with a combined score, was established to uncover the CTgenes.

To determine the number of the CTgenes, a set of core genes is required to compare with the test genes. The core genes were defined to be significantly associated with cold tolerance or response to cold stress in soybean. Criteria for core genes selection were as follows: (1) the most significantly reported genes that were associated with cold tolerance or response to cold stress; (2) the most frequently reported genes with significant characteristics (i.e. reported on more than three layers, combined score ranked within the top 0.5% of the test genes, and scored higher than 3 at each layer excluding homologs); and (3) only genes that performed transcriptome measurement and were further validated by the quantitative polymerase chain reaction (q-PCR) with significant *p*-value less than 0.0001. An optimal cut-off score between the core genes (a left-skewed distribution) and the test genes (a right-skewed distribution) was identified to determine the number of the CTgenes in the NPRF prioritization procedure.

### The NPRF prioritization

We proposed the NPRF prioritization algorithm based on the decision tree model to select important genes for feature fitness iteratively. A bootstrap resampling and random permutation approach were applied to build *r* decision trees from bootstrap samples. The final decision can be classified according to the votes of the *r* trees. We defined the test genes as the original training set $D=\left\{\left(G_{j},S_{j}^{l}\right)|j=1,\cdots ,n;l=1,\cdots m\right\}$ , where *G* represents a gene in the test genes and *S^l^* represents a score on the *l*-th layer. That is, the original training set *D* contains *n* genes, and there are *m* scores in each gene. The procedures of the NPRF prioritization algorithm (please see the ‘Non-Parameter RF Prioritization’ panel in **Figure 3**) are elaborated as follows.

Step 1. Generating *r* bootstrap samples. We randomly generated *r* (10,000 times, say) bootstrap samples (denoted as $D_{t}^{bt}$ , *t*=1,⋯,*r* ) of the same size of *n* from the original training set *D* (i.e. test genes) with replacement. We defined $D^{bt}=\left\{D_{1}^{bt},D_{2}^{bt},\cdots ,D_{r}^{bt}\right\}$ as a collection of *r* bootstrap samples for training tree models. The top *h* genes were determined by selecting the top *h* with the highest combined scores. We denoted $D_{t,top}^{bt}=\left\{\left(G_{tj'}^{bt},S_{tj'}^{bt}\right)|j'=1,\cdots ,h\right\}\;\left(t=1,\cdots ,r\right)$ as the top set for bootstrap sample.

Step 2. Generating *q* permutation samples. To compare with a certain bootstrap sample, a null model was used to randomly shuffle scores in order to break the structure inherent in the original training set *D*. As a result, *q* (10,000 times, say) permutation samples (denoted as $D_{t'}^{perm}$ , *t*'=1,⋯,*q* ) were constructed, and here we defined $D^{perm}=\left\{D_{1}^{perm},D_{2}^{perm},\cdots ,D_{q}^{perm}\right\}$ as a collection of *q* permutation samples. For each $D_{t'}^{perm}$ (*t*'=1,⋯,*q* ), we selected the top *h* genes with the highest combined scores. Similarly, we denoted $D_{t',top}^{perm}=\left\{\left(G_{t'j'}^{perm},S_{t'j'}^{perm}\right)|j'=1,\cdots ,h\right\}\;(t'=1,\cdots ,q$ ) as the top set for *q* permutation samples.

Step 3. Constructing *r* decision tree models. For each bootstrap sample, the combined scores of the top bootstrap genes ( $S^{bt}=\left\{S_{j'}^{bt}|j'=1,\cdots ,h\right\}$ ) were compared with those of *q* sets of the top permutation genes ( $S^{perm}=\left\{S_{1j'}^{perm},S_{2j'}^{perm},\cdots ,S_{qj'}^{perm}|j^{\prime }=1,\cdots ,h\right\}$ ) using the Wilcoxon rank-sum test to construct a decision tree model. A decision tree model was built only if the combined scores of the top bootstrap genes were significantly distributed higher than those of the top permutation genes. Otherwise, we discarded the decision tree, and regenerated a bootstrap sample followed by the permutation process to construct a new decision tree model until a total of *r* decision tree models were collected.

Step 4. Ranking *r* decision trees to build a final model. We pooled all the *r* decisions trees (the top bootstrap genes), and ranked them by the frequency of counts for each gene. The final decision tree model was identified by selecting the top *h* with the highest counts (this is the well-established CTgenes).

### Validation studies of the CTgenes

The whole RNA-seq database of cold-treated leaves of soybean seedlings (Yamasaki and Randall, 2016) was used as an independent sample to validate the reliability and robustness of the CTgenes. Cold experiments on 2-week-old soybean (c.v. Williams 82) seedlings were treated at 4 °C for 2 days after the light was operated for 4 hours on day 10, and maintained at 4 °C under the light-dark cycles till harvest time (0, 1, and 24 hours). All experiments were performed in triplicate. Because of the comprehensive and large-scale of databases, their results were frequently used to confirm soybean’s physiological experiments (Robison et al., 2019; Li et al., 2022). Robison et al. (2019) reviewed Yamasaki and Randall's results (2016) and conducted RNA-seq analysis to confirm the ethylene-mediated signaling pathway has negative impacts on CBF/DREB-regulated cold responses in soybean underlying different scenarios of cold treatments. Li et al. (2022) later conducted genome-wide analysis of homologs using proteins sequences to identify mitochondrial calcium uniporter family genes, and validated by RT-PCR assays under cold treatment (harvested and measured gene expression after 0, 1, and 24 hr cold treatment at 4°C), which echo Yamasaki and Randall's results (2016) results . These evidenced that the whole genome RNA-seq databases can be a basis of cold-tolerant responses in soybean for further validation, evaluation, and experiments. We employed the ‘edgeR’ package in R to quantify the relative expression level of transcripts from RNA-seq data (https://bioconductor.org/packages/release/bioc/html/edgeR.html). In total, 49,778 gene expression data from RNA-seq were generated for validation studies.

Three approaches were applied to validate the CTgenes using the RNA-seq data. Statistical methods and sampling techniques were devised as follows. First, we compared our CTgenes with the remaining genes. The Wilcoxon rank-sum test was used to verify whether the CTgenes had smaller *p*-values than the remaining genes. Second, we compared our CTgenes with random gene sets. For simplicity, the process of the sampling approach is described below. (1) Sampling: we randomly sampled a set of genes (same size as the CTgenes) without replacement from the gene pool of the expression data. (2) Statistical testing: the CTgenes were compared to the random set using the Wilcoxon rank-sum test. (3) Loop: we repeated the above steps until 10,000 random sets were obtained. (4) Calculating an empirical *p*-value: we counted the frequency of random sets that outperformed the CTgenes divided by 10,000 to obtain an empirical *p*-value. Third, we performed the Wilcoxon rank-sum test and the hypergeometric test to compare our CTgenes with other candidate genes identified from other methods, including the RF prioritization on Rafsee (Zhai et al., 2016), the step-function adjusted factor-based (SFAF) prioritization (Lai et al., 2021), the network-based prioritization on SoyNet (Kim et al., 2017), candidate genes selection on SoyBase (https://www.soybase.org/), and the QTLs mapping approach (Jiang et al., 2009; Qiu et al., 2011; Zhang et al., 2012). All statistical resampling and analyses were implemented *via* Python 3.8 version. For detailed framework of validation, please refer to **Figure 3** (please see the ‘Prediction & Validation’ panel).

### Pathway enrichment and crosstalk network analysis

To better understand the whole map of the molecular mechanisms underlying cold stress in soybeans, we introduced systems biology approaches to computationally examine the CTgenes in the manner of comprehensive framework and systematical thinking. The GO is a comprehensive database for soybeans, which integrates abundant terms (13,292 GO terms) on the functions of genes. The GO annotations provide links between genes and biological processes, cellular components, or molecular functions. In this study, we conducted pathway enrichment analysis to investigate physiological and biological pathways that are overrepresented in cold tolerance or response to cold stress. The hypergeometric test was applied to test enrichment for genes in a specific pathway (i.e. GO term) against genes outside the pathway, using the CTgenes and the GO terms. Pathways whose gene numbers were greater than 2,500 or smaller than 5 were excluded from the analysis to prevent overly limited information or excessively large pathways. All *p*-values were adjusted by Bonferroni correction to account for false positive results.

To visualize module discovery, we conducted network analysis through a pathway crosstalk to understand alternative information between biological functions of complex systems underlying cold tolerance in soybean. To explore the pathway crosstalk, we calculated the degree to describe the connections of a node in the crosstalk network. A pathway crosstalk network consists of nodes (i.e. biological pathways) and edges (i.e. overlapping genes between pathways), which is widely used to describe communications or interactions between functional pathways. Here we defined the node color, node size, and edge width to present the complicated relations between biological functions in an information-enriched way. The node size was defined as the significance level of a certain pathway from the hypergeometric test. The edge width was defined as the overlapping genes between pathways. The node color was used to distinguish short- (purple), mid- (yellow), and long-term (purple) cold tolerance. Edges in pink, gold, and blue connect short-, mid-, and long-term pathways, respectively. Edges in gray represent connections between mid- and long-term pathways.

### Validation in soybean samples

A total of 56 soybean samples were used to conduct cold tolerance experiments (unpublished data) to evaluate cold resistant and susceptible varieties. We investigated soybeans response to cold stress at the V3 stage by recording brown spots, curl, wrinkled on leaves and plant development after low temperature occurrence (the minimum air temperature below 10°C). All samples were genotyped using the Axiom^®^ 180K SoyaSNP array. Fifty-five SNPs located in the CTgenes were selected for cluster analysis. SNPs were used as genotypic data for assessing relationships among soybean germplasms. A distance matrix was calculated as identity-by-state dissimilarity using PLINK software (Purcell et al., 2007), and a phylogenetic tree was constructed using the neighbor-joining tree method in MEGA X (Kumar et al., 2018). The neighbor joining tree was visualized by Interactive Tree of Life available at https://itol.embl.de/. We performed a cluster analysis on soybean samples to investigate whether the CTgenes are able to distinguish cold tolerance and susceptible varieties in soybean.

### Implementation environment

All the analyses were implemented on the Dell PowerEdge R930 4-socket 4U rack server model that supports four processors based on the Intel^®^ Xeon^®^ CPU E7-4830-V3 2.10GHz. This server consists of 64-core CPUs, 512G RAM, and 110TB SAS HD memory. All the analyses were operated in both Python v3.8 and R Linux 64-bit v4.2.1. The gene network analyses of the selected CTgenes were employed on the SoyNet (https://www.inetbio.org/soynet/) to obtain a network edge information, followed by the Cytoscape (https://cytoscape.org/) to create and visualize the functional modules.

## Results

### Literature study

We found three articles using the linkage mapping approach to identify the candidate QTLs relevant to cold tolerance in soybean (Jiang et al., 2009; Qiu et al., 2011; Zhang et al., 2012). These QTLs were collected in the DNA layer. In total, 11 articles conducted the expression profiles on targeted genes in soybean under low temperature, including genes encoding *GmFLC-like* protein (Lyu et al., 2020), CBF/DREB1 transcription factors (Robison et al., 2019), *GmIRCHS* genes (Ohnishi et al., 2011), heat shock transcription factors (Chung et al., 2013), genes encoding CCA1-like proteins (Bian et al., 2017), *HSP*20 gene family (Lopes-Caitar et al., 2013), RCC1 gene family (Dong et al., 2021), 14-3-3 gene family (Wang et al., 2019), carboxylase gene family (Wang et al., 2016), histone deacetylases gene family (Yang et al., 2018), and genes encoding NIMA-related kinase 1 (GmNEK1) (Pan et al., 2017). In addition, five articles were collected on the basis of a non-coding RNA molecular approach, including circRNA (Wang et al., 2020b) and miRNA (Maruyama et al., 2012; Zhang et al., 2014; Xu et al., 2016; Sun et al., 2020), to uncover their impact to other transcriptional RNA under cold stress in soybean. These genes were collected in the RNA layer. The PPIN database demonstrates the functional relationships between gene pairs, which was downloaded from the PlantRegMap (http://plantregmap.cbi.pku.edu.cn/). These gene pairs were collected in the protein layer. Three articles using the pathway regulation methods were collected, of which two were classified into the RNA layer (Yamasaki et al., 2013; Yu et al., 2014) and the other was in the function layer (Tian et al., 2015a). The genetic data were involved with several pathways that are relevant to cold-tolerant mechanisms in soybean.

### OnO data mining and collection

We developed a systems biology pipeline, including data input (iterative OnO data wrangling and data-ensemble), data processing (OnO data integration and gene prioritization), output (CTgenes discovery, enriched pathways, and module discovery), and validation in soybean samples (cold tolerance experiments) to achieve the robustness and reliability of the CTgenes (**Figure 1**). We applied keyword search to screen relevant OnO data related to cold tolerance or response to cold stress in soybean (**Figure 2**). A total of 65 publications and 5 databases were collected initially. After being carefully examined by well-trained and experienced experts, 22 articles and 3 databases were relevant to this study and hence collected in the OnO data integration. As a result, 54 QTLs were curated from 3 articles in the DNA layer. There is no any data from pathway analysis in the DNA layer. Nine QTLs (features of mRNA) and 12,441 genes (features of circRNA and miRNA) were collected from 16 articles and 1 database in the RNA layer. Among them, only one omics data set (containing 12,343 genes) was included in the cold experiments on 2-week-old soybean seedlings (cv. Nourin No. 2) at 4 °C for 1 day. 47,931 protein pairs were mined from 717,676 PPIN of the PlantRegMap (http://plantregmap.cbi.pku.edu.cn/download.php#networks) database in the protein layer. In addition, 74 genes were extracted from 2 pathways (ABA biosynthetic process and ABA catabolic process) that related to cold tolerance in the GENEONTOLOGY (GO; http://geneontology.org/) pathway regulation database. We excavated 992 genes in pathway regulations (features of metabolites) from one article in the metabolome layer. In the homologs layer, a total of 1,800 (*Arabidopsis*) and 317 (*Medicago*) journal articles related to cold treatment were initially collected from NCBI PubMed. Articles that involved irrelevant or not applicable to the present study (i.e. non-significant results, lack of experimental validation, not focused on cold tolerance experiments, and genes validated in other plants) were discarded. As a result, 395 and 11 articles were retained, resulting in 608 *Arabidopsis* genes and 45 *Medicago* genes, respectively. These *Arabidopsis* and *Medicago* genes were separately mapped to 3929 and 31 gene homologous in soybean. In total, 65,452 genetic data were obtained from the OnO data. For detailed information on OnO data collection, please refer to **Tables 1A**, **B**. **Supplementary Material 1** provides an overview of collected OnO data. Each genetic data was classified into different biological layers according to the types of features and the analytic approaches available in each OnO data layer.

**Table 1A:** The summary of the collected genetic data. (A) The number of non-omics and omics (OnO) data collected from articles and databases. (B) Summary information of collected OnO data from different molecular approach.

OnO data	Layer/Feature		Approach		No. of articles/databases mined initially	No. of articles/databases after quality check			No. of genetic data (SNP, gene, SSR, QTL) collected	
Genome	DNA/QTLs		Linkage mapping		9/0	3/0			54	
			Pathway analysis		3/0	0/0			0	
Transcriptome	RNA/mRNA		Gene expression		35/1	11/0			9	
	RNA/circRNA		Noncoding RNA		1/0	1/0			26	
	RNA/miRNA		Noncoding RNA		4/1	4/1			12,415	
	RNA/mixed		Pathway regulation		8/2	2/1			74	
Proteome	Protein/Protein		PPIN		0/1	0/1			47,931	
Metabolome	Metabolome/Metabolites		Pathway regulation		5/0	1/0			992	
Total					65/5	22/3			61,492	

SNP, single nucleotide polymorphism; QTL, quantitative trait locus; mRNA, messenger RNA; circRNA, circular RNA; miRNA, micro RNA; PPIN, protein-protein interaction network.

**Table 1B:** 

**OnO data**		**Layer / Feature/ Approach**		**Growing stage**			**Cold treatment**	**Reference**		**Duration of cold tolerance**
**Omics data:**										
Transcriptome		RNA / miRNA / Noncoding RNA		2-weeks-old			4 °C for 1 day	Maruyama et al. (2012)		Mid-term
**Non-omics data:**										
Genome		DNA / QTL / Linkage mapping		Started at 2 days after sowing			6 °C treatment for 8 days	Qiu et al. (2011)		Long-term
		DNA / QTL / Linkage mapping		Germination stage			6 °C treatment for 1 week	Zhang et al. (2012)		Long-term
		DNA / QTL / Linkage mapping		Started at 5 days after sowing			6 °C treatment for 1 week	Jiang et al. (2009)		Long-term
Transcriptome		RNA / mRNA / Gene Expression		In second trifoliate			15 in daytime and 13 °C at night for 10 days	Lyu et al. (2020)		Long-term
		RNA / mRNA / Gene Expression		1-week-old			18 in daytime and 13 °C at night for 14 days	Ohnishi et al. (2011)		Long-term
		RNA / mRNA / Gene Expression		10-days-old			4 °C for 12 hr	Yang et al. (2018)		Mid-term
		RNA / mRNA / Gene Expression		3~4-weeks-old			4 °C for 0, 3, 6, 24 hr	Chung et al. (2013)		Long- & short-term
		RNA / mRNA / Gene Expression		4-weeks-old			4 °C for 1, 6, 12 hr	Wang et al. (2019)		Short-term
		RNA / mRNA / Gene Expression		10-days-old			4 °C for 24 hr	Bian et al. (2017)		Mid-term
		RNA / mRNA / Gene Expression		Growing stage V3			4 °C for 3 hr	Lopes-Caitar et al. (2013)		Short-term
		RNA / mRNA / Gene Expression		10-days-old			4 °C for 0, 1, 24 hr	Robison et al. (2019)		Mid- & short-term
		RNA / mRNA / Gene Expression		3-weeks-old			4 °C for 6, 12 hr	Dong et al. (2021)		Short- & mid-term
		RNA / mRNA / Gene Expression		2-weeks-old			4 °C for 3, 6, 12 hr	Pan et al. (2017)		Short- & mid-term
		RNA / mRNA / Gene Expression		4-weeks-old			4 °C for 3, 6, 12, 24 hr	Wang et al. (2016)		Short- & mid-term
		RNA / circRNA / Noncoding		Under trifoliate fully expanded			4 °C for 0, 4, 8 hr	Wang et al. (2020b)		Short-term
		RNA / miRNA / Noncoding		One true-leaf stage			4 °C for 24 hr	Xu et al. (2016)		Mid-term
		RNA / miRNA / Noncoding		20-days-old			0 °C for 2, 12 hr	Sun et al. (2020)		Short- & mid-term
		RNA / miRNA / Noncoding		4-week-old			4 °C for 24 hr	Zhang et al. (2014)		Mid-term
		RNA / mixed / Pathway regulation		10-days-old			4 °C for 2 days	Yamasaki et al. (2013)		Long-term
		RNA / mixed / Pathway regulation		10-days-old			4 °C for 0, 12, 24, 48 hr	Yu et al. (2014)		Mid-term
Proteome		Protein / Protein / PPIN		Database from PlantRegMap (http://plantregmap.cbi.pku.edu.cn/)						
Metabolome		Function / Metabolites / Pathway regulation		Complete expansion of the first trifoliate			5 °C for 0, 12, 24 hr	Tian et al. (2015a)		Mid-term

PPIN, protein-protein interaction networks.

### Integrated feature selection and feature extraction

**Figure 4:**
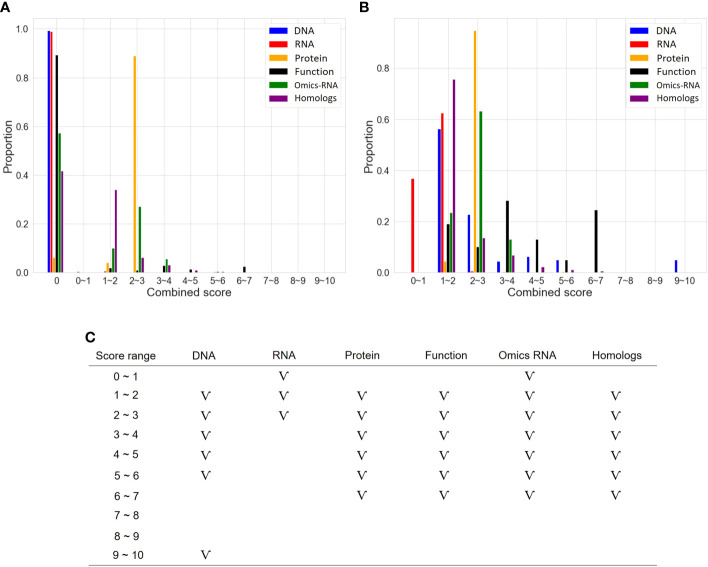
The distribution of scores in six layers. **(A)** Distribution of categorized scores in six layers. **(B)** Distribution of categorized scores (excluding 0 scores) in six layers. **(C)** The pattern of categorized scores (excluding 0 scores) in six layers. The symbol ‘V’ represents the existence of genes in the score range.

As described in the “MATERIALS AND METHODS” section, many steps are executed in the OnO data-ensemble processes to minimize potential noise and biases (**Figure 3**). There were 44 articles and 2 databases of unwanted data (i.e. irrelevant to this study). Hence, we removed them from OnO data integration in the data cleaning step (**Table 1**). In order to unify distinct data sources, all collected OnO data were correspondingly transformed into limited scores ranging from 0 to 10 (**Figure 4A**) to account for data heterogeneity in the data harmonization step (**Supplementary Table 1**). Finally, 64 QTLs and 47,931 protein pairs were respectively mapped into 449 and 46,770 genes in the identifiers mapping step. Taken together, 60,726 genes with combined scores were extracted from integrated OnO data. Most of these scores were bounded between 0 to 7, and skewed to the right in each of all layers (**Figures 4B, C**). It is clear to note that many genes played no role in the cold tolerance or response to cold stress (that is, scored 0 across all layers); thus, they were excluded from the analyses. As a result, a total of 4,014 (short-term), 14,607 (mid-term), and 4,069 (long-term) genes were retained for gene prioritization algorithm. Here, we denoted these genes as the test genes for short-, mid-, and long-term cold tolerance.

### The NPRF prioritization

**Figure 5:**
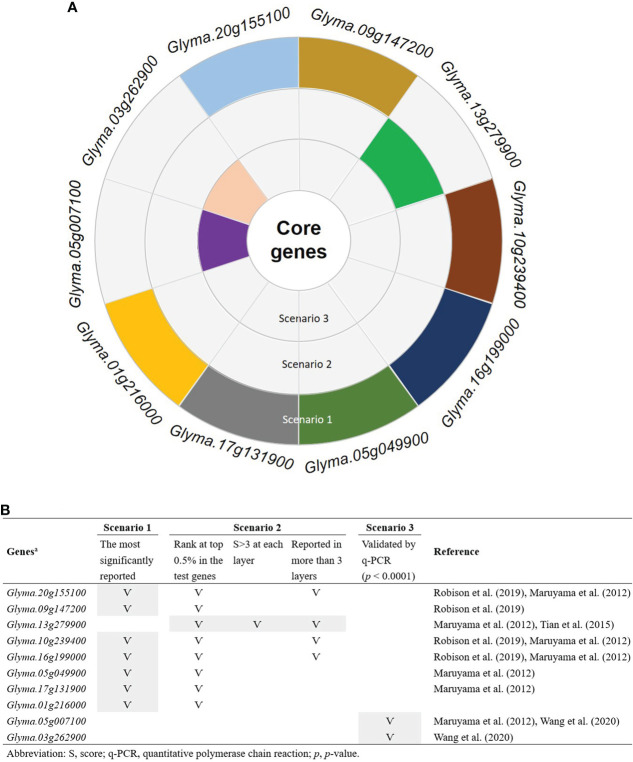
Ten selected core genes. **(A)** The sunburst chart of the selection of the core genes. **(B)** The selection criteria of the core genes. Only genes that meet at least one of the three scenarios were selected as the core genes. Scenario 1: the most significantly reported genes that were associated with cold tolerance or response to cold stress. Scenario 2: the most frequently reported genes with significant characteristics. Scenario 3: only genes that performed transcriptome measurement were further validated by the quantitative polymerase chain reaction (q-PCR) with significant *p*-value less than 0.0001.

**Figure 6:**
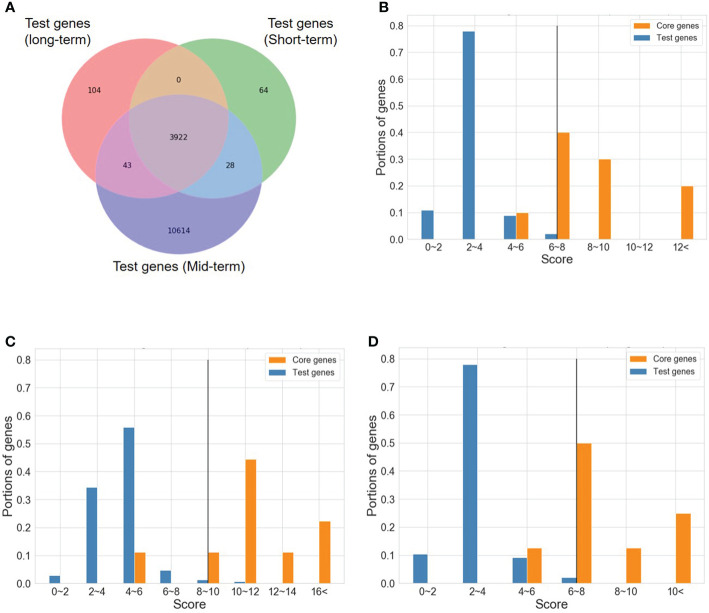
A cut-off score between the core genes and the test genes. **(A)** The Venn diagram of test genes among short-, mid-, and long-term. **(B)** A cut-off score between the core genes and the test genes for short-term CTgenes. **(C)** A cut-off score between the core genes and the test genes for mid-term CTgenes. **(D)** A cut-off score between the core genes and the test genes for long-term CTgenes.

**Figure 7:**
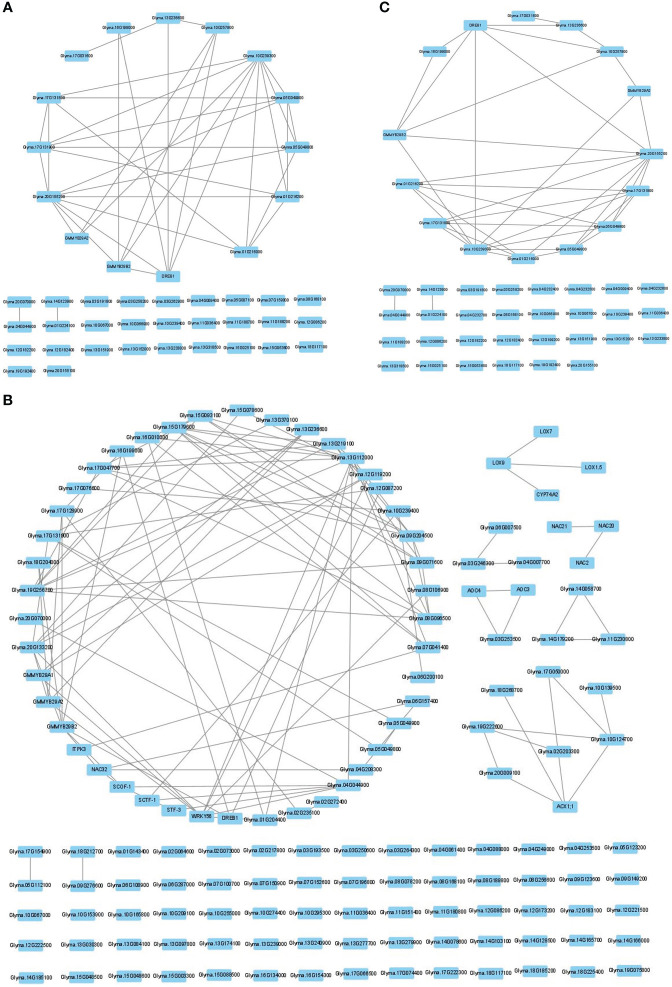
The CTgenes discovery for short-, mid-, and long-term cold tolerance. A total of 44, 143, and 45 prioritized CTgenes were identified for **(A)** short-term, **(B)** mid-term, and **(C)** long-term cold tolerance.

**Figure 8:**
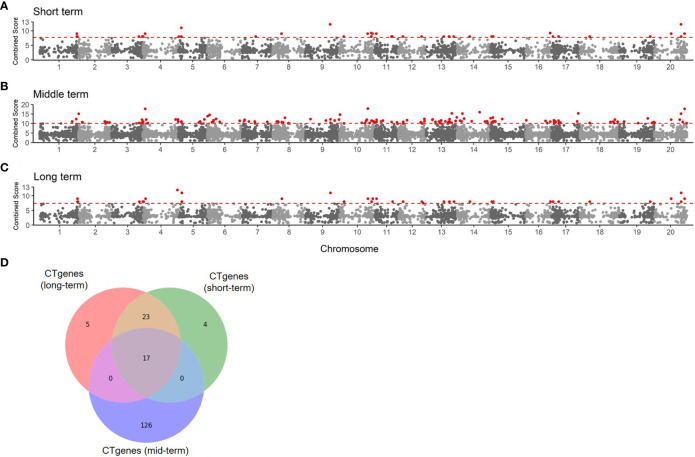
The Manhattan plot of the test genes for **(A)** short-term, **(B)** mid-term, and **(C)** long-term. Dots colored in red are the CTgenes. **(D)** The Venn diagram of CTgenes among short-, mid-, and long-term.

To determine the number of CTgenes, we carefully selected ten core genes (**Figure 5A**), which met one of the three scenarios (**Figure 5B**) (see the “MATERIALS AND METHODS” section), and compared them with the test genes. The overlapping genes among these test genes are demonstrated in **Figure 6A**. A clear separation at a cut-off score of 8 (**Figure 6B**), 10 (**Figure 6C**), and 7 (**Figure 6D**) was observed between the core genes and the test genes, determining the number of CTgenes to prioritize from the test genes for short-, mid-, and long-term cold tolerance was 44, 143, and 45, respectively. The feature fitness of the integrated OnO data was implemented through the NPRF prioritization algorithm. We performed bootstrapping to create a bootstrap sample, and tested it on 10,000 permutation samples using the Wilcoxon rank-sum test. This procedure was repeated until 10,000 sets of CTgenes were identified. We then counted the number of times each gene was selected as the prioritized CTgenes. For each gene, the gene count was divided by 10,000 to obtain the gene probability. After removing genes with zero counts, the gene probabilities for short-, mid-, and long-term were ranged between 0.0404-1.0 (101 genes), 0.0001-1.0 (190 genes), and 0.0411-1.0 (102 genes), respectively (Supplementary material 2). We prioritized them and found a dramatically drop at the 45^th^ (declines from 0.8974 to 0.0525), 144^th^ (declines from 0.5077 to 0.2575), and 46^th^ (declines from 0.5215 to 0.0531) gene in gene probabilities for short-, mid-, and long-term gene set, respectively. As a result, a total of 44, 143, and 45 prioritized CTgenes were identified for short- (**Figure 7A**), mid- (**Figure 7B**), and long-term (**Figure 7C**) cold tolerance, respectively. Of which, one major module was found in short- (15 CTgenes), mid- (44 CTgenes), and long-term (15 CTgenes) cold tolerance CTgenes. The Manhattan plots of the test genes for short- (**Figure 8A**), mid- (**Figure 8B**), and long-term (**Figure 8C**) cold tolerance were demonstrated, where the CTgenes were colored in red dots. Seventeen and twenty-three CTgenes overlapped among the three terms and among short and long term, respectively (**Figure 8D**). The most clustering of the CTgenes on chromosome 10 (6, 12, and 6 genes in short-, mid-, and long-term, respectively) and 13 (5, 15, and 5 genes in short-, mid-, and long-term, respectively) were identified; however, no any CTgenes on chromosome 2 and 6 were noticed for both short- and long-term cold tolerance in soybean.

### Validation studies of the CTgenes

**Figure 9:**
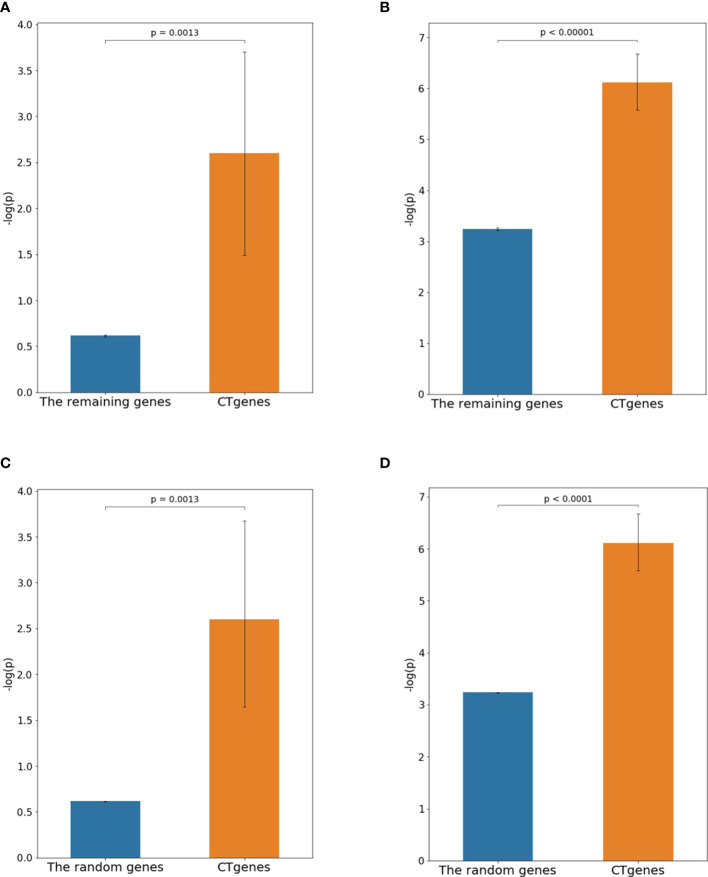
Validation studies of the CTgenes compared to the remaining genes and random genes using an independent omics data. **(A)** Comparing short-term CTgenes with the remaining genes (1hr cold treatment). **(B)** Comparing mid-term CTgenes with the remaining genes (24hr cold treatment). **(C)** Comparing short-term CTgenes with the random genes (1hr cold treatment). **(D)** Comparing mid-term CTgenes with the random genes (24hr cold treatment).

The independent RNA-seq data (Yamasaki and Randall, 2016) was applied to examine the reliability of the CTgenes. Data of 1-hour and 24-hour cold exposure; were applied to test for short- and mid-term respectively. First, we compared the CTgenes with the remaining genes (excluding the CTgenes). Our CTgenes had significantly smaller *p*-values than the remaining genes for short (*p*-value =1.3×10^-3^) and mid-term (*p*-value<1.0×10^-5^) cold tolerance, respectively (**Figures 9A, B**). Second, we compared the CTgenes with 10,000 sets of random genes (some of the CTgenes may be included by chance) sampled from the test genes set. Similarly, our CTgenes outperformed the random genes for short- (**Figure 9C**) and mid-term (**Figure 9D**) cold tolerance (*p*-values ranged from 0.0013 to<1.0×10^-4^).

**Figure 10:**
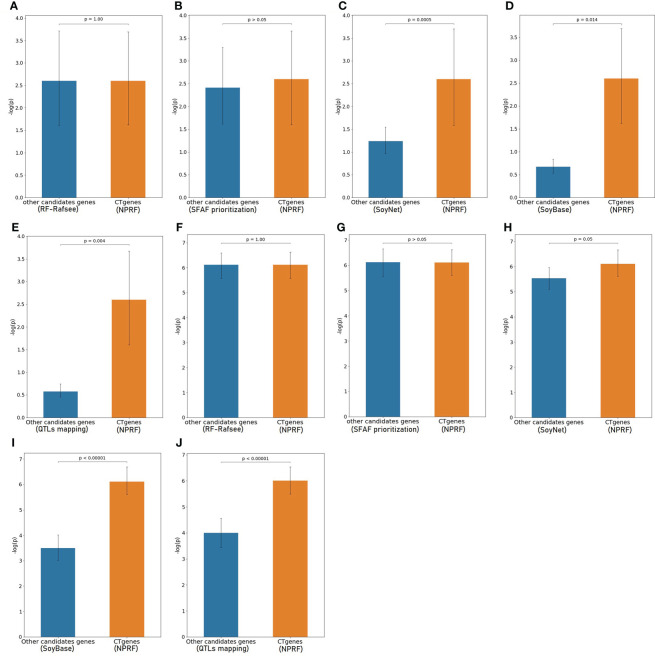
Validation study of the CTgenes compared to the candidate genes identified by other methods using an independent omics data. Comparing short-term (1 hr cold treatment) CTgenes with candidate genes identified by **(A)** the RF prioritization, **(B)** the SFAF prioritization, **(C)** the network-based prioritization on SoyNet, **(D)** candidate genes selection on SoyBase, and **(E)** the QTLs mapping approach. Comparing mid-term (24hr cold treatment) CTgenes with candidate genes identified by **(F)** the RF prioritization, **(G)** the SFAF prioritization, **(H)** the network-based prioritization on SoyNet, **(I)** candidate genes selection on SoyBase, and **(J)** the QTLs mapping approach.

To evaluate the robustness of our CTgenes identified through the OnO integration and the NPRF prioritization algorithm, we further compared them with other cold tolerant candidate genes selected by other methods (e.g. the RF prioritization on Rafsee, the SFAF prioritization, the network-based prioritization on SoyNet, candidate genes selection on SoyBase, and the QTLs mapping approach). First, we compared our NPRF results with the results of the RF prioritization on Rafsee, and found that both methods produced identical short- (**Figure 10A**), mid- (**Figure 10F**), and long-term CTgenes (*p*-values =1). Second, we conducted the SFAF weighting scheme to evaluate all collected genes, and observed that 24 (54.5%), 118 (82.5%), and 23 (51.1%) overlapped short-, mid-, and long-term CTgenes between the SFAF prioritization and the NPRF prioritization, respectively. Although non-significant differences were observed among two prioritized top genes in short- (*p*-values =0.79) (**Figure 10B**) and mid-term (*p*-values =0.37) (**Figure 10G**) cold tolerance, the joint effect analyses of the hypergeometric test were quite different. Only four enriched pathways, including JA biosynthetic process, response to fungus, response to JA stimulus, and response to wounding (*p*-values <1.0×10^-16^), were reported in the mid-term cold tolerance in both approaches. The difference of the prioritized top genes in the SFAF prioritization from our CTgenes was significantly enriched in several pathways, including 2 pathways (cold acclimation and regulation of GA biosynthesis) in the short-term, 4 pathways (JA biosynthetic process, response to fungus, root meristem growth, and root system development) in mid-term, and 1 pathway (cold acclimation) in long-term cold tolerance; however, the difference of our CTgenes from the prioritized top genes in the SFAF prioritization were found only 2 enriched pathways (JA biosynthetic process and vitamin metabolic process) relevant to the mid-term cold tolerance. Third, we compared our CTgenes to 221 candidate genes identified by network-based prioritization in SoyNet (https://www.inetbio.org/soynet/search.php), and found our CTgenes significantly performed better (*p*-values ranged from 0.05 to <1.0×10^-5^) than prioritized genes in SoyNet for short- (**Figure 10C**), and mid-term (**Figure 10H**) cold tolerance. Fourth, a total of 272 genes mapped from 66 SSRs were reported to be related to soybean cold stress in SoyBase (https://www.soybase.org/). We compared our CTgenes with the 272 genes, and found our CTgenes performed significantly better (*p*-values ranged from 0.014 to <1.0×10^-5^) than the 272 genes discovered in SoyBase, for short- (**Figure 10D**) and mid-term (**Figure 10I**). Finally, our short- (**Figure 10E**), and mid-term (**Figure 10J**) CTgenes outperformed 134 candidate genes identified in QTLs (Jiang et al., 2009; Qiu et al., 2011; Zhang et al., 2012) from the linkage mapping approach (*p*-values ranged from 0.004 to <1.0×10^-5^). The results further elucidate that our CTgenes performed equally well with the RF prioritization and outperformed other approaches. This suggests that the CTgenes identified by our systematic pipelines are reliable and robust, and have the potential to uncover novel biological pathways and physiological mechanisms underlying cold tolerance in soybean.

### Pathway enrichment analysis

**Figure 11:**
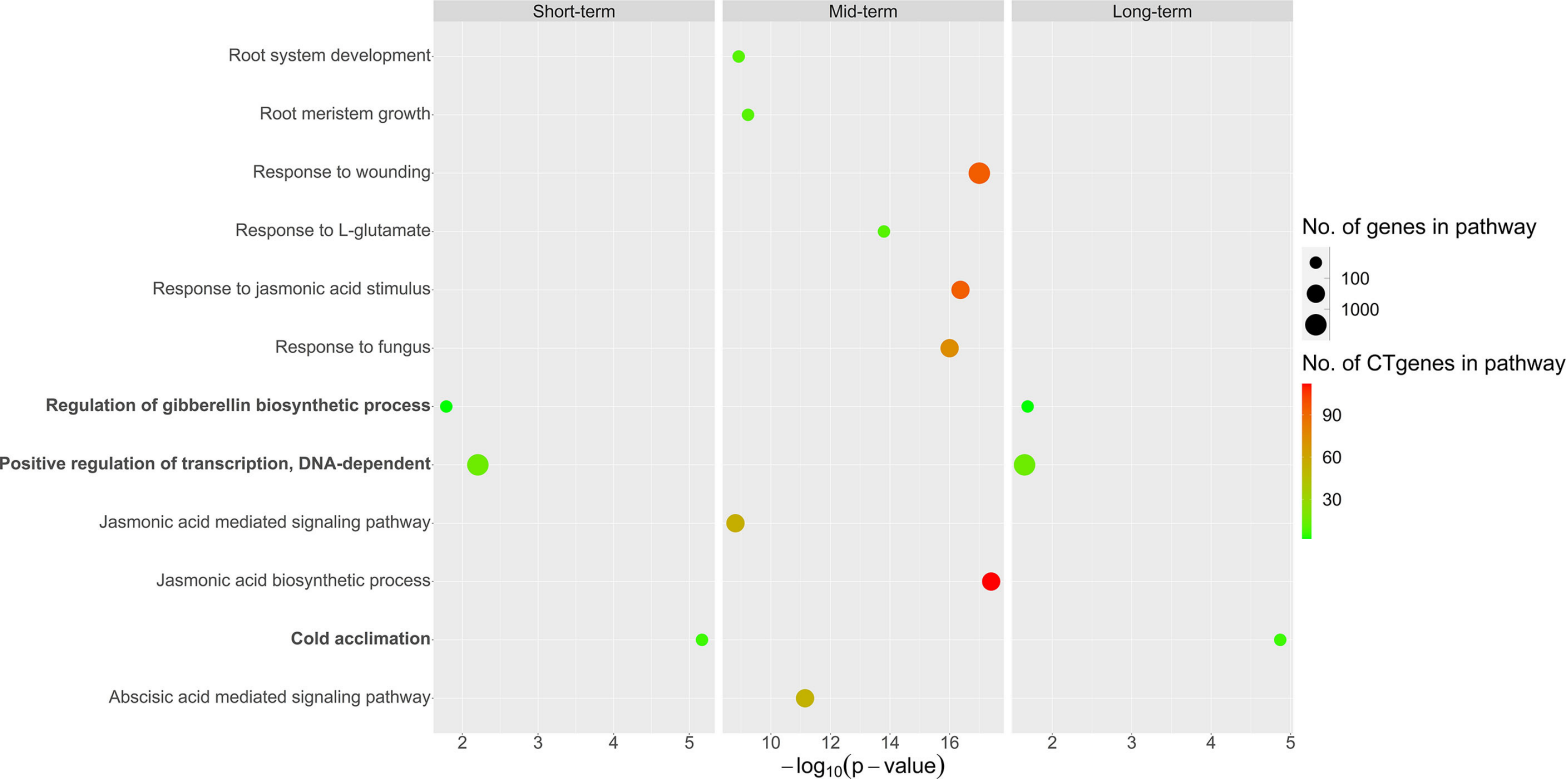
Pathway enrichment analysis of the CTgenes for short-, mid-, and long-term cold tolerance.

Overall, our CTgenes were significantly enriched in 12 GO pathways (3 in short-term, 9 in mid-term, and 3 in long-term). Among them, three pathways (regulation of gibberellin (GA) biosynthetic process, positive regulation of transcription, DNA-dependent, and cold acclimation) were found in both short- and long-term. Nine pathways were solely identified in the mid-term, where the top four pathways were JA biosynthetic process, response to fungus, response to JA stimulus, and response to wounding. Detailed information on enriched pathways of the CTgenes for short-, mid-, and long-term, please refer to **Figure 11** and **Supplementary Table 3**.

### Module discovery

**Figure 12:**
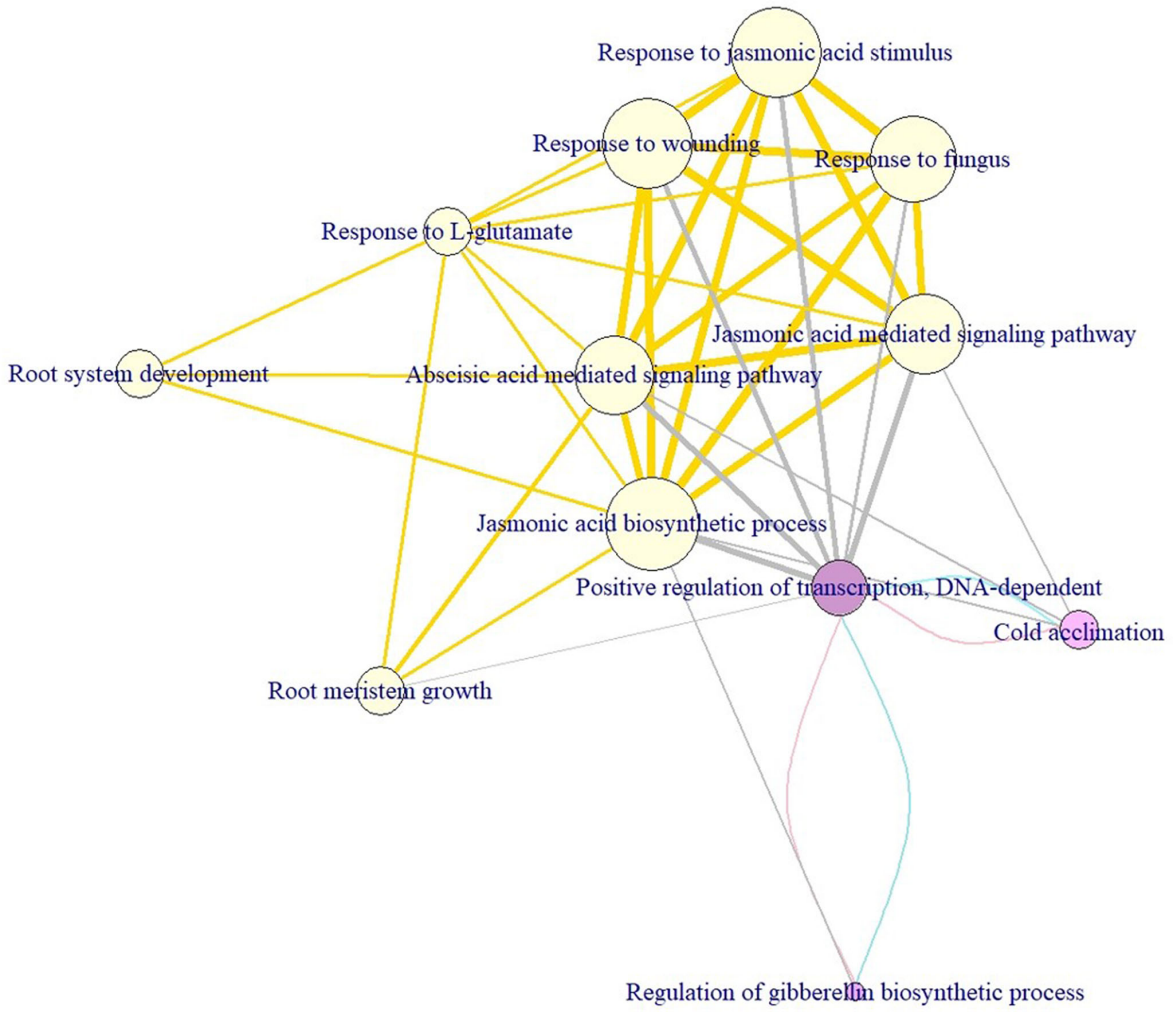
Module discovery underlying the crosstalk of enriched pathways and functional network of the CTgenes for short-, mid-, and long-term cold tolerance. This crosstalk consists of 12 enriched pathways (3 from short-term, 9 from mid-term, and 3 from long-term cold tolerance) and edges (i.e. overlapping genes between 2 linked pathways, or pathway crosstalk). The node size was defined as the significance level of a certain pathway from the hypergeometric test. The edge width was defined as the overlapping genes between pathways. The node color was used to distinguish short- (purple), mid- (yellow), and long-term (purple) cold tolerance. Edges in pink, gold, and blue connect short-, mid-, and long-term pathways, respectively. Edges in gray represent connections between mid- and long-term pathways.

To deeply understand how the enriched pathways are related to cold tolerance, we constructed the crosstalk network to uncover the phenomenon of interaction or cooperation between pathways. The average (median) degree of the pathways for the short-, mid-, and long-term cold tolerance was 6.33 (5), 7 (7), and 6.33 (5), respectively. Non-significant differences in degree values were observed between the three terms (*p*-values >0.05), indicating the complex interactions among the CTgenes and the pathways were similar in the three terms. **Figure 12** demonstrates the pathways crosstalk and functional map of the CTgenes. In the short-term cold tolerance, three enriched pathways formed a module (pink nodes), which was dominated by response to stimulus (e.g. cold acclimation) and primary metabolic process (e.g. regulation of GA biosynthetic process and positive regulation of transcription, DNA-dependent). In the mid-term cold tolerance, nine pathways formed a module (gold nodes), which were relevant to the primary metabolic process (e.g. JA biosynthetic process), response to stimulus (e.g. ABA-mediated signaling pathway, JA-mediated signaling pathway, response to JA stimulus, response to L-glutamate, response to wounding, and response to fungus), and system development (e.g., root meristem growth and root system development). The long-term cold tolerance showed an identical module to the short-term. Taken together, these 12 enriched pathways formed one self-clustered module dominated by hormone-related and defense-related pathways.

### Validation in soybean samples

**Figure 13:**
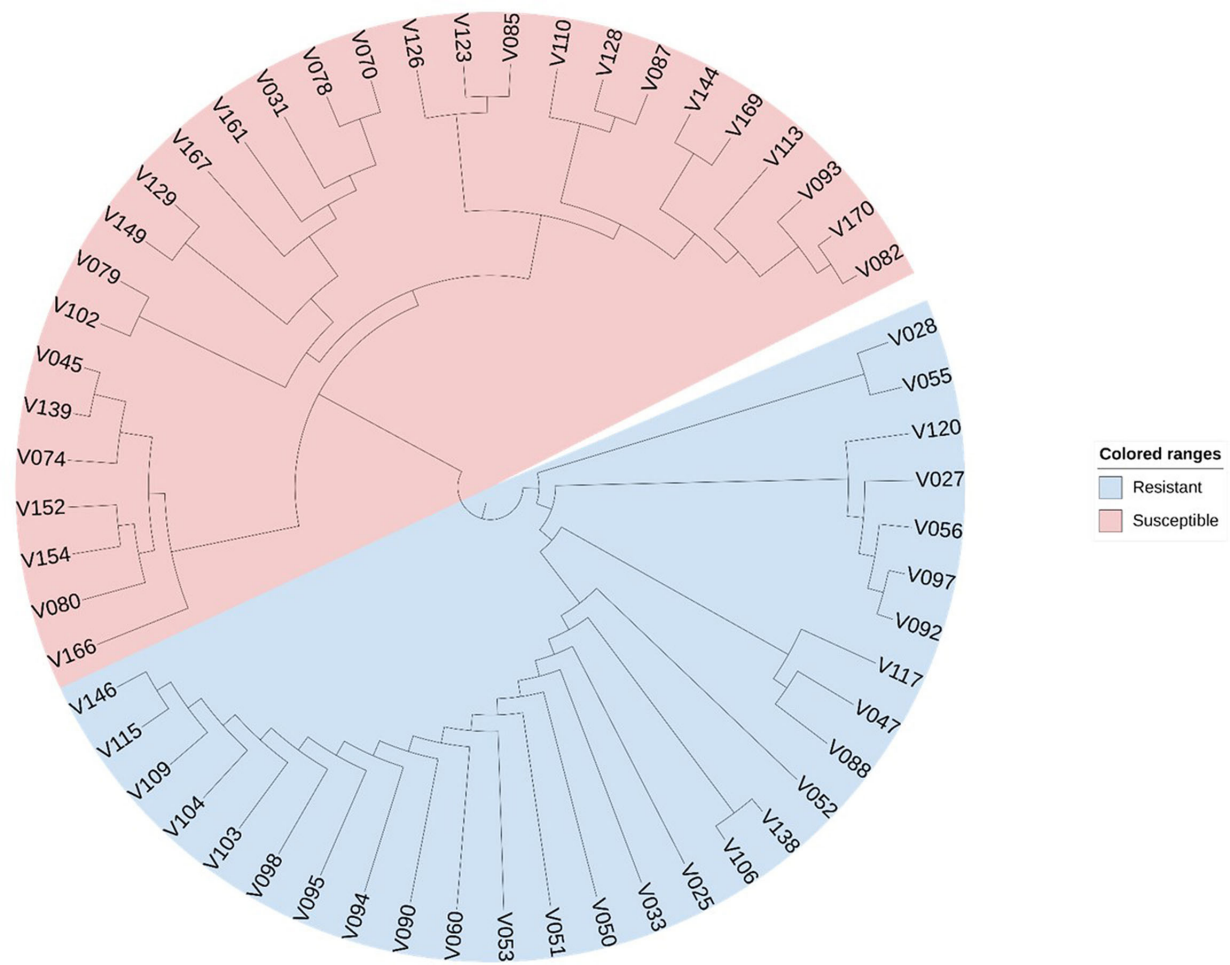
Sample classification using the CTgenes. A total of 39 CTgenes were used to conduct cluster analysis. The neighbor-joining algorithm was performed to construct phylogenetic trees. As a result, 56 soybean varieties were classified into two groups (resistant lines vs. susceptible lines) according to their genotype patterns. The blue color represents cold tolerant varieties. The red color represents cold susceptible varieties.

We selected 56 soybean varieties to validate the effectiveness of the CTgenes selection. Of which 28 varieties are resistant to low temperature, and the remaining are susceptible, based on soybean cold-treatment experiments. All samples were genotyped using the SoyaSNP180K chip array. We pooled all the CTgenes across all three terms, and obtained 91 CTgenes after discarded genes with no SNPs information. Furthermore, we conducted SNP-gene mapping and removed SNPs only when all samples received the same genotype. As a result, 39 CTgenes (including 55 SNPs) were retained for sample classification in cluster analysis. **Figure 13** demonstrated a clear separation between soybean samples, suggesting our CTgenes can distinguish 56 soybean varieties into 28 cold-resistant (colored in blue) and 28 cold-susceptible (colored in red) varieties.

## Discussion

Cold tolerance in plants is a complex abiotic trait, requiring a good understanding of the mechanisms behind the complex biological system. It is also important to take into account the interactions (i.e. joint effects) with other factors like environmental, ecological, and other plant hormones (e.g. auxins) (Ishibashi et al., 2013). There are numerous studies investigating genetic information on cold tolerance or cold response from the level of DNA, RNA, and protein to function. In this regard, great efforts in systematically analyzing these multi-dimensional data are needed to uncover insight into biologically meaningful contexts. In the present, we developed a comprehensive framework through systematic strategies on OnO data mining, data-ensemble, gene prioritization, and external independent validation to select the prioritized CTgenes from the collected gene pool that are relevant to cold tolerance or response to cold stress. This study proposes a systems biology framework to bridge the knowledge gap between genetic information about cold tolerance or cold response and the OnO data.

To the best of our knowledge, this is the first work on the OnO data integration from different molecular layers and gene prioritization for the discovery of soybean cold-tolerant genes (i.e. CTgenes), followed by enriched pathways, module discovery (i.e. a combination of similar functions regarding the underlying cold tolerance), validation in soybean samples (i.e. cold tolerance experiments), and sample classification (i.e. resistant vs. susceptible varieties) to address the robustness and effectiveness of the CTgenes selection. This work’s challenges involved interconnections, heterogeneity, noise, and high-dimensional feature profiles across different layers of the OnO data. There have been several attempts to eliminate or minimize the impact of these issues by the data-ensemble step on improving data quality.

Rapid progress in high-throughput technologies has booted advances in plant omics. Several collections of omics, including genomics, transcriptomics, proteomics, and metabolomics, have become available for crop improvement. Unfortunately, deriving biological insights from a single layer of omics data is often limited, although it may explain some specific phenomena biologically (Cao et al., 2022). In this case, it may be difficult to gain knowledge from the results of single omics to apply directly to plant breeding. Recently, several reviews have emphasized the importance of multi-omics data integration for obtaining a comprehensive view underlying a complex trait to provide reliable biological insight (Pazhamala et al., 2021). In soybeans, genomics and transcriptomics have developed as expected, but the progress of proteomics and metabolomics still dropped behind (Deshmukh et al., 2014). Initially, we found four collections of transcriptomics databases related to cold-treated experiments in soybeans, including 3 RNA-seq databases and one microarray database. Among them, two RNA-seq databases were discarded due to being genetically-modified and irrelevant to the target of this study. Only two omics data were relevant to soybean cold tolerance (Maruyama et al., 2012; Yamasaki and Randall, 2016), pointing out the situation and bottleneck of current plant omics integration. To cope with such a situation, we extend the idea of multi-omics techniques to integrate omics and non-omics data in the same models, as proposed by López De Maturana et al. (2019), to complete the whole contour of the cold-tolerant mechanism to a large extent.

In the present study, we combined different types of the OnO features in different molecular layers through DNA, RNA, protein, function, and homologs (**Table 1B**). Typically, different layers have distinct features, with potential interactions between and within them. In addition, each of the OnO data was generated from different experimental designs, analytic methods, environmental factors, and cultivars, bringing much more challenges to integrative analysis. To systematically analyze the OnO data, we proposed the systems biology strategy to effectively pool, integrate, and analyze diverse data formats and varying data types (*p*-value, LOD, FC, degree, cluster coefficient, and score) underlying different statistical approaches and biological objectives. Data heterogeneity is typically a challenging task in the OnO data integration. In this study, the association-based scoring system was developed to unify distinct data types generated by varied technologies in different layers. Most of the genes were scored between 0 to 6 (more than half of gene scores were ranged between 0-2) in each layer to prevent overestimation of scoring. Furthermore, a rigorously multi-staged data quality control process was also implemented in the data-ensemble step (containing data clean, data harmonization, data heterogeneity, and data mapping) to remove unwanted data, false positives, and noise, so that the risk of overestimation, uncertainties, and false positive results can be effectively minimized. This can provide accurate, valid, and reliable results, in a comprehensive and consistent manner.

The degree of injury caused by low-temperature in plants varies from different cold-treated spans. Considering global climate changes in soybean-producing areas, soybean cold tolerance can be categorized into short-, mid-, and long-term according to periods of cold exposure and damages to soybean plants. Radiative cooling leads to rapid temperature drops at midnight is typically the scenario for a short-term chilling environment. A sudden period of chilling or brief exposure to low-temperature will not pose a serious threat to plant physiological mechanisms, and plants still survive. Consecutive low temperature lasting 2 days in winter’s subtropical area belongs to the mid-term cold stress. Long-lasting (more than 2 days) cold spell and unusual cold extreme climate represents the long-term cold stress. Prolonged low-temperature stress, however, may increase the accumulation of toxic substances in plant tissue, which not only seriously influences the photosynthesis and other metabolic pathways, but also results in some unfavorable phenomena, such as chlorosis, necrosis, wilting, and even death (Lukatkin et al., 2012; Adam and Murthy, 2014). The longer in cold environments, the more damaging to the plants’ physiology. Hence, to precisely define the CTgenes, information on cold-treated span, and temperature were included in the models to classify these OnO data into short-, mid-, and long-term cold tolerance groups.

The selection of the CTgenes is a challenging task, as we first need to know how many genes are in the collection. To determine the number of CTgenes, ten core genes (**Figure 5**) were selected to compare to the test genes (i.e. the training set) to determine the required number of CTgenes. The central idea is to use combined-scores skewness to determine the optimal cut-off point in separating two score distributions between the core genes (skewed to the left) and the test genes (skewed to the right). Eight core genes (*Glyma.20g155100*, *Glyma.09g147200*, *Glyma.13g279900*, *Glyma.10g239400*, *Glyma.16g199000*, *Glyma.05g049900*, *Glyma.17g131900*, and *Glyma.01g216000*) and two core genes (*Glyma.05g007100* and *Glyma.03g262900*) were ranked within the top 0.5% and 1.7-10% of the test genes, respectively. Among them, *Glyma.20g155100* (GmDREB1B;1), *Glyma.09g147200* (GmDREB1A;1), *Glyma.10g239400* (GmDREB1B;2), *Glyma.16g199000* (GmDREB1A;2), *Glyma.05g049900* (GmDREB1D;1), *Glyma.17g131900* (GmDREB1D;2), and *Glyma.01g216000* (GmDREB1C;1) are the *CBF*/*DREB1*s genes. Several studies mentioned that GmDREB proteins in soybean play a central role in cold tolerance mechanisms (Yamasaki and Randall, 2016; Robison et al., 2019). Furthermore, some studies claimed that *CBF*/*DREB1*s genes acted as transcription factors in *Arabidopsis* resistance to cold stress (Fowler and Thomashow, 2002; Zhao et al., 2016). Therefore, in this study, we evidenced that precisely selecting the core genes can improve the discovery of biomarkers (i.e. CTgenes).

The distributions of combined scores of the core genes and the test genes differed (please refer to **Figure 6**). A gene-threshold for the combined score was chosen to obtain good discriminability in separating the core gene set from the total test genes to select final CTgenes. We conducted the hypergeometric test to identify enriched pathways for assessing the gene-threshold selection in the systems biology framework. We selected three different gene-thresholds (the lower bound, the middle point, and the upper bound) from a cut-off bin. For instance, a cut-off score of 6 (the lower bound), 7 (the middle point), and 8 (the upper bound) was used to select the 200, 100, and 44 CTgenes in short-term cold tolerance, which resulted in 23, 9, and 3 enriched pathways that relevant to cold-tolerant responses, respectively. Among them, 2 pathways (“positive regulation of transcription, DNA-dependent” and “cold acclimation”) were reported in three scenarios. Similar situations can be found in the long-term CTgenes. For the mid-term CTgenes, a cut-off score of 8 (the lower bound), 9 (the middle point), and 10 (the upper bound) were used to select the 331, 205, and 143 CTgenes, resulting in 36, 29, and 9 enriched pathways that relevant to cold-tolerant responses, respectively. Among them, all 9 pathways (“root system development”, “root meristem growth”, “response to wounding”, “response to L-glutamate”, “response to jasmonic acid stimulus”, “response to fungus”, “jasmonic acid mediated signaling pathway”, “jasmonic acid biosynthetic process”, and “abscisic acid mediated signaling pathway”) were overlapped in the three gene-threshold scenarios. It is not surprising that the more CTgenes resulted in the more enriched pathways, where the more false-positive results might be included. This suggests that the CTgenes selected through a rigorous gene-threshold using the upper bound in the cut-off bin demonstrated power to uncover enriched pathways. Hence, the upper bounded gene-threshold in the cut-off bin can be regarded as the optimal gene-threshold for CTgenes selection. It can be used as a reference for the physiological effects and biological mechanisms of important crop traits by comparing multiple cut points in the same bin to screen out important biological pathways.

Gene prioritization is often challenging given the large-sized and high-dimensional OnO data in the analytical space and the complex trait of cold tolerance in nature. Another challenge is efficiently dealing with uncertainties, false positives, and noisy data and accurately select valuable characteristics and meaningful information from such massive amounts of big data. The NPRF prioritization algorithm has been proposed to address these issues for the integrated OnO data. For a specific marker, we hypothesized that it is less likely to include noisy data and false positive results in each of all layers. The more molecular layers included in integrated OnO data, the smaller the chance of resulting in false positive results. In our prioritization algorithm, the features in each layer were scored separately, and then all distinctive features across different molecular layers were merged to a reduced space (i.e. a single combined score) for dimensionality reduction without losing the algorithm’s accuracy. This NPRF prioritization algorithm constructed a decision tree through selecting the most important features from the test genes set to account for uncertainties at each bootstrap iteration. The feature stability was assessed by gene-probability of the selected features over 10,000 bootstrap iterations. As a result, a small collection of prioritized genes was respectively selected as the CTgenes for short-, mid- and long-term cold tolerance in soybean (**Figures 7**-**9**). The short- (**Figure 7A**), mid- (**Figure 7B**), and long-term (**Figure 7C**) CTgenes all revealed one primary module based on topological characteristics (i.e. degree), which was involved with response to cold, cold acclimation, and response to freezing. These CTgenes were related to plant hormones (JA, GA, ABA, and ethylene) and defense-related pathways. In addition, several gene groups related to plant hormones were identified in mid-term CTgenes. The group containing *LOX7*, *LOX9*, *LOX1.5*, and *CYP74A2* was also related to the defense response pathways. The group of *Glyma.06g007500*, *Glyma.03g246300*, and *Glyma.04g007700* were related to the defense system and cold-related response (**Figure 7B**).

**Figure 14:**
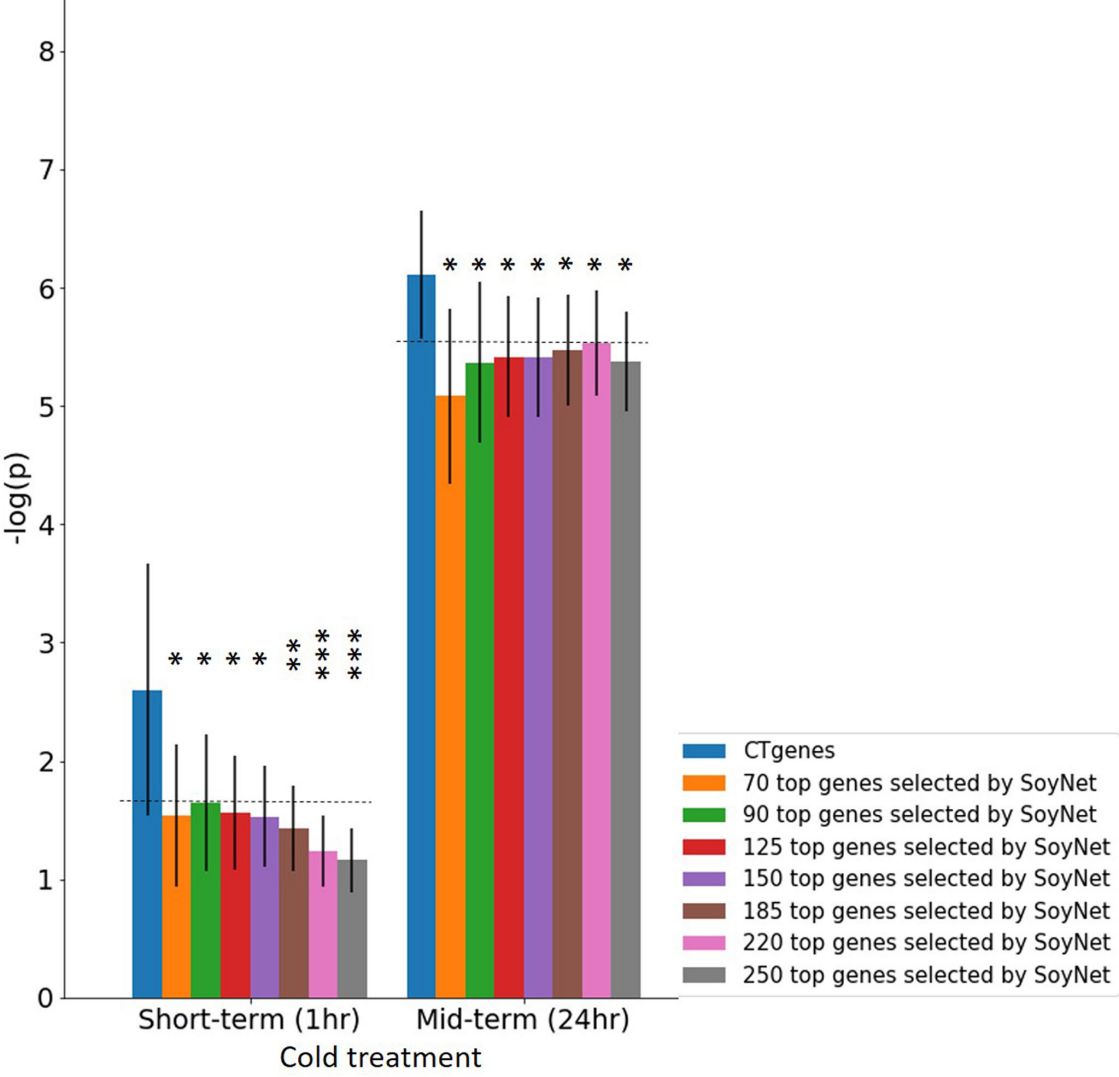
Validation study of the CTgenes compared to the candidate genes identified by SoyNet using an independent omics data. A range of different numbers (70, 90, 125, 150, 185, 220, and 250) of top genes were used to evaluate the robustness and the reliability of the CTgenes selected through our comprehensive and systematic framework. The horizontal line above the bar chart is the threshold of statistical significance level (* < 0.05, ** < 0.01, *** < 0.001).

Our CTgenes were compared to the remaining genes (i.e. non-CTgenes), random selected genes (**Figure 10**), and other candidate genes identified by other existing methods (i.e., the RF-Rafsee, the SFAF prioritization, the network-based prioritization on SoyNet, candidate genes selection on SoyBase, and the QTLs mapping approach) (**Figure 11**), and validated in an independent RNA-seq database to prove the effectiveness, stability, and reliability of the selected features following the schema illustrated in **Figure 3**. Overall, our results indicated that the CTgenes selected from integrated OnO data and the NPRF prioritization had superior performance to the ones identified from a single or a few layers and most other existing methods. The NPRF prioritization and the RF-Rafsee both produced the same top genes and performed equally well (**Figures 10A, F**). Supplementary material 2 provided detailed information about the gene-probabilities of the top genes identified by the RF-Rafsee method. No statistically significant difference typically refers to the difference not exceeding a particular threshold value. In the validation study, we observed a non-significance difference between the NPRF prioritization and the SFAF prioritization (**Figures 10B, G**); however, this did not mean there is no biological meaning. Therefore, we further conducted the hypergeometric test to systematically examine the joint effect of the difference among two top genes identified from both methods. Most interestingly, the different sets of prioritized top genes identified by the SFAF prioritization from our CTgenes (denoted as CTgenes^NPRF\SFAF^) had the power to uncover enriched pathways relevant to the cold-tolerant responses in all three cold treatments (**Supplementary Table 4**). However, the different sets of our CTgenes from prioritized top genes identified in the SFAF prioritization (denoted as CTgenes^SFAF\NPRF^) only found enriched pathways in mid-term (**Supplementary Table 4**). This indicates that our CTgenes selected by the NPRF prioritization had more power to uncover the mechanisms underlying the cold-tolerant responses in soybean. To further validate the robustness of our CTgenes, we compared our CTgenes to a range of the top genes (70, 90, 125, 150, 185, 220, 250) identified from network-based prioritization in SoyNet. **Figure 14** demonstrated that our CTgenes significantly outperformed all ranges of the SoyNet top genes in an independent RNA-seq database, suggesting the robustness and the reliability of the CTgenes selected through our comprehensive systems biology-based framework.

Our CTgenes were significantly enriched in 12 GO pathways (**Figure 12**). Cold acclimation is a natural mechanism for the plants of temperate origins. However some subtropical or tropical species, such as soybean, may also have excellent mechanisms to acclimate to cold (Cabané et al., 1993), which may enhance tolerance to cold treatment. The levels of phenolic acids in soybean seedlings will decrease significantly during cold acclimation, which modifies cell wall extensibility to adapt to a chilling environment. GA can facilitate soybean seedlings’ emergence and increase the shoot height under cold stress (Wang et al., 1996), which plays a key role in regulating soybean seed germination under low temperature. Moreover, GA is an important regulator in other species (e.g., Arabidopsis and cotton) to cope with low temperatures (Achard et al., 2008). JA is one of the main plant growth regulators, acting antagonistically in regulating of plant immune and development, and acts as a pivotal role in many abiotic stresses (Wang et al., 2020a). JA can prevent reactive oxygen species (ROS) formation by enhancing chilling tolerance (Sharma and Laxmi, 2016). Studies relevant to the interaction between JA and cold tolerance were reported in some species such as sweet orange (Habibi et al., 2019), but not in soybean. In Arabidopsis, JA regulates the CBF/DREB1 factors and activates the CBF/DREB1-independent pathway to enhance cold tolerance (Hu et al., 2013). In this study, three JA related pathways (response to JA, JA mediated signaling pathway, and JA biosynthetic process) were found to be significantly enriched with cold tolerance or response to cold stress. To date, this is the first work to address the interaction between JA and cold tolerance in soybean. Fungi play an important role in conferring abiotic stress tolerance in plants, for instance, cold resistance in soybean (Begum et al., 2019). Some particular bacteria and fungi can facilitate the physiological mechanism in plants under environmental stress (Levy et al., 1983). Arbuscular mycorrhizal fungi (AMF) is the common symbiotic fungus, forming a symbiosis with 80% of plant species (Smith and Read, 2008), which can improve cold resistance under low temperatures in maize. In soybean, AMF-inoculated commercial cultivar showed better shape with higher leaf area and yield without stress treatment, than non-inoculated one (Adeyemi et al., 2020). Thus, soybean cold tolerance under cold stress may benefit from AMF symbiosis. More work is remained to physiologically evaluate their connections in soybean. ABA is also one of the key hormones to react to abiotic stress in plants. The application of ABA enhances the cold resistance in crop species, such as rice (Tian et al., 2015b). A recent study verified that the exogenous ABA could induce the GmABI3 (ABSCISIC ACID INSENSITIVE 3), and further activate the ABA-dependent protein to confront the cold stress in soybean (Manan and Zhao, 2020). Interaction between ABA and JA signaling pathways can synergistically enhance the resistance to abiotic stress (Manan and Zhao, 2020). It is worth noting that our CTgenes prioritized from the integrated OnO data boost the power of their potential roles in uncovering meaningful results, some with biological novelties, for studying the molecular mechanisms of cold tolerance in soybean through pathway enrichment analysis.

To better understand the CTgenes pattern, a total of 40 CTgenes were selected from 12 enriched GO pathways. We analyzed these genes in the SoyBase ‘Gene Model Data Mining and Analysis’ tool, and found that they were related to response to cold (21 genes), cold acclimation (3 genes), response to freezing (2 genes), response to temperature stimulus (1 gene), response to osmotic stress (3 genes), response to stress (5 genes), immune-related (7 genes), and defense response (27 genes) related pathways (**Supplementary Table 2**). Interestingly, both cold acclimation and response to osmotic stress pathways shared the same CTgenes having major role in response to cold stress, of which *Glyma.15g048600* (homologs of *At4g08500* in Arabidopsis) was enriched in response to cold, cold acclimation, response to osmotic stress, immune-related, and defense response-related pathways, indicating this novel gene may play an important role in the complex mechanisms underlying cold-tolerant in soybean.

Multiple mechanisms involved in cold-tolerant or response to cold stress were revealed across various plant species (Lukatkin et al., 2012). However, understanding of the molecular mechanisms underlying cold tolerance in soybean still remains limited and unclear. Pathway crosstalk networks provide in-depth knowledge of the whole picture of cold-tolerant mechanisms in soybean. Our pathway crosstalk network (**Figure 12**) revealed two clustered modules, both of which had important implications for cold tolerance. In soybean, GA- and ABA-mediated pathways are found to be involved with cold tolerance (Wang et al., 1996; Manan and Zhao, 2020); however, both JA- and fungi response pathways have not been reported to be involved with the cold-tolerant mechanisms previously. Evidence showed that acclimation to low temperature (Cabané et al., 1993), activating plant hormones biosynthesis to alleviate the negative impact of chilling environment and the growth induction of roots (Janas et al., 2000) were the primary strategies in soybeans at low temperatures. Our pathway crosstalk network (**Figure 12**) demonstrated the whole map of cold-tolerant soybean, which were classified into 3 modules, including response to stimulus, metabolic process, and system development, showing interactions between hormones- and defense-related pathways underlying cold environment. More specifically, cold acclimation shared several genes with JA-mediated signaling pathway, and JA- and ABA-mediated signaling pathways involved with many overlapping genes. These results were accorded with the previous studies (Hu et al., 2013; Wang et al., 2020a).

The selected CTgenes were used for sample classification. A total of 56 varieties (28 cold resistant varieties and 28 susceptible varieties) were used to evaluate the effectiveness and reliability of the CTgenes. Phenotypic information was collected from soybean cold tolerance experiments during the spring crop season in 2020 and 2022. A completely randomized design with ten replicates was performed in field experiments. We investigated soybeans’ response to cold stress (minimum air temperature below 10°C at the V3 stage) by recording brown spots, curl, wrinkled on leaves, and plant development after low temperature occurrence. The resistance and susceptibility of the whole panel were used in cold tolerance experiments as the test results. The result of sample classification (**Figure 13**) suggested that our CTgenes have the power to distinguish resistant and susceptible lines, which strongly supported the effectiveness and reliability of the CTgenes.

This study developed the novel concept of integrating multiple OnO data systematically and comprehensively way to assess insight into the physiological mechanisms in soybean cold tolerance. Additionally, the proposed NPRF gene prioritization method can evaluate the importance of each gene based on the physiological knowledge, offering more informative results. Nonetheless, there were three limitations in the present study. First, the reliability of our CTgenes was based on the OnO data integrity. Although we integrated nearly all articles about soybean cold tolerance, some uncertainties still existed, including noise, biases, and outdated data. Fortunately, we not only do prudent data quality control, but also fully employ data clean, data harmonization, data heterogeneity, and data mapping across the data-ensemble step to deal with such problems nicely. Second, the progress in the soybean omics field has developed as expected only for genomics and transcriptomics. However, the progress of proteomics and metabolomics still drops behind (Deshmukh et al., 2014). To date, we only found 2 omics data. One omics data was integrated into the OnO data, and the other served as independent omics data for the validation sample. Therefore, it may get caught into difficulty to demonstrate the comprehensiveness of omics data in soybean. To cope with it, during OnO data integration process, we also took the non-omics data into account to increase the precision of biomarkers discovery and the phenotype prediction. Third, the process of data integration is inevitable to face the risk of false positives. As known, false positive results often exist in many previously reported results. We hypothesized that false positives would not occur in all different layers by chance. In the present study, we employed the high dimensional OnO data integration and gene mapping approach to scoring genes across different layers to efficiently minimize false positives. By overcoming such limitations, we can successfully present the complete contour of the cold-tolerant mechanism in soybean to the extent.

## Conclusion

This study shed new light on the effectiveness of the CTgenes prioritized from integrated OnO data and provided a systems biology pipeline for uncovering the mechanisms behind cold tolerance in soybean. We developed a computational systems biology framework to eliminate the impact of uncertainties and false positives, so that the CTgenes can be precisely selected without loss of information. The CTgenes demonstrated great power to uncover enriched pathways and the mechanisms, and module discovery. Our framework exhibited the powerful potential to identify novel biomarkers and their underlying molecular pathways or mechanisms, providing novel insights into the response to cold stress. Most importantly, our CTgenes were validated in cold tolerance field trials, suggesting the reliability and effectiveness of the selection of the CTgenes.

With an increasing severity and frequency of cold extremes, the growth, quality, and yield of soybean are negatively affected by biotic and abiotic stresses, usually in combination. Hence, there is an urgent need to discover key genes to enhance cold tolerance in soybeans. The CTgenes and relevant biological analysis results provide some molecular insights and future application directions. First, our CTgenes have demonstrated good discriminability in separating the resistant varieties from the susceptible ones, which can be widely applied to be the basis of further soybean molecular biology research, such as cold-related or cross-resistant experiments. Second, the systems biology pipelines proposed in this study offer great potential in crop research to boost the breeding program of new resistant soybean cultivars with durable resistance to cold stress, bringing forward the new cultivars to overcome the direr climate change. Third, the proposed framework in the present study could be applied to other important traits of interest in soybean and extended to other model plant species to adapt to changing environments for improvements in agricultural productivity.

## Data availability statement

The data presented in the study are deposited in the DRYAD repository, accession number for a unique digital object identifier (DOI): doi: 10.5061/dryad.b5mkkwhgp.

## Author contributions

C-FK conceived the study conception and design. P-HK, Z-YL, C-JL, and Y-SL collected the data. C-FK, P-HK, SB, L-HJ, and C-MH performed the analysis and interpreted the data. P-HK, C-FK, C-MH, and L-HJ drafted manuscript. C-FK and P-HK revised manuscript. All authors contributed to the article and approved the submitted version.

## Funding

This work was supported by grant 2022-CCASF-Agr-5 from Chung Cheng Agriculture Science and Social Welfare Foundation. This work was financially supported (in part) by the Advanced Plant Biotechnology Center from The Featured Areas Research Center Program within the framework of the Higher Education Sprout Project by the Ministry of Education (MOE) in Taiwan.

## Acknowledgments

We thank Mu-Chien Lai, Shih-Hsun Hung, Guan-Lin Chuo, and Chi-Wei Hsieh for plant physiology, molecular biotechnology insight, and programming knowledge support. We also thank Min-Lun Lee for English editing.

## Conflict of interest

The authors declare that the research was conducted in the absence of any commercial or financial relationships that could be construed as a potential conflict of interest.

## Publisher’s note

All claims expressed in this article are solely those of the authors and do not necessarily represent those of their affiliated organizations, or those of the publisher, the editors and the reviewers. Any product that may be evaluated in this article, or claim that may be made by its manufacturer, is not guaranteed or endorsed by the publisher.

## References

[B1] Achard P. Gong F. Cheminant S. Alioua M. Hedden P. Genschik P. (2008). The cold-inducible CBF1 factor–dependent signaling pathway modulates the accumulation of the growth-repressing DELLA proteins *via* its effect on gibberellin metabolism. Plant Cell 20, 2117–2129. doi: 10.1105/tpc.108.058941 18757556 PMC2553604

[B2] Acharjee A. Kloosterman B. Visser R. G. Maliepaard C. (2016). Integration of multi-omics data for prediction of phenotypic traits using random forest. BMC Bioinform. 17, 363–373. doi: 10.1186/s12859-016-1043-4 PMC4905610 27295212

[B3] Adam S. Murthy S. (2014). “Effect of cold stress on photosynthesis of plants and possible protection mechanisms,” in Approaches to plant stress and their management. Eds. Gaur R. K. Pradeep S. (India: Springer).

[B4] Adeyemi N. O. Atayese M. O. Olubode A. A. Akan M. E. (2020). Effect of commercial arbuscular mycorrhizal fungi inoculant on growth and yield of soybean under controlled and natural field conditions. J. Plant Nutr. 43, 487–499. doi: 10.1080/01904167.2019.1685101

[B5] Aerts N. Pereira Mendes M. Van Wees S. C. (2021). Multiple levels of crosstalk in hormone networks regulating plant defense. Plant J. 105, 489–504. doi: 10.1111/tpj.15124 33617121 PMC7898868

[B6] Bandara A. Y. Weerasooriya D. K. Bell T. H. Esker P. D. (2021). Prospects of alleviating early planting-associated cold susceptibility of soybean using microbes: New insights from microbiome analysis. J. Agron. Crop Sci. 207, 171–185. doi: 10.1111/jac.12476

[B7] Begum N. Qin C. Ahanger M. A. Raza S. Khan M. I. Ashraf M.. (2019). Role of arbuscular mycorrhizal fungi in plant growth regulation: Implications in abiotic stress tolerance. Front. Plant Sci. 10. doi: 10.3389/fpls.2019.01068 PMC6761482 31608075

[B8] Bian S. Jin D. Li R. Xie X. Gao G. Sun W.. (2017). Genome-wide analysis of CCA1-like proteins in soybean and functional characterization of GmMYB138a. Int. J. Mol. Sci. 18, 2040. doi: 10.3390/ijms18102040 PMC5666722 28937654

[B9] Breiman L. (2001). Random forests. Mach. Learn. 45, 5–32. doi: 10.1023/A:1010933404324

[B10] Cabané M. Calvet P. Vincens P. Boudet A. M. (1993). Characterization of chilling-acclimation-related proteins in soybean and identification of one as a member of the heat shock protein (HSP 70) family. Planta 190, 346–353. doi: 10.1007/BF00196963 7763662

[B11] Camacho C. Coulouris G. Avagyan V. Ma N. Papadopoulos J. Bealer K.. (2009). BLAST+: architecture and applications. BMC Bioinform. 10, 1–9. doi: 10.1186/1471-2105-10-421 PMC2803857 20003500

[B12] Cao P. Zhao Y. Wu F. Xin D. Liu C. Wu X.. (2022). Multi-omics techniques for soybean molecular breeding. Int. J. Mol. Sci. 23, 4994. doi: 10.3390/ijms23094994 35563386 PMC9099442

[B13] Chung E. Kim K.-M. Lee J.-H. (2013). Genome-wide analysis and molecular characterization of heat shock transcription factor family in *Glycine max* . J. Genet. Genom. 40, 127–135. doi: 10.1016/j.jgg.2012.12.002 23522385

[B14] Deshmukh R. Sonah H. Patil G. Chen W. Prince S. Mutava R.. (2014). Integrating omic approaches for abiotic stress tolerance in soybean. Front. Plant Sci. 5. doi: 10.3389/fpls.2014.00244 PMC4042060 24917870

[B15] Ding Y. Shi Y. Yang S. (2019). Advances and challenges in uncovering cold tolerance regulatory mechanisms in plants. New Phytol. 222, 1690–1704. doi: 10.1111/nph.15696 30664232

[B16] Dong Z. Wang H. Li X. Ji H. (2021). Enhancement of plant cold tolerance by soybean RCC1 family gene GmTCF1a. BMC Plant Biol. 21, 1–16. doi: 10.1186/s12870-021-03157-5 34384381 PMC8359048

[B17] Fowler S. Thomashow M. F. (2002). Arabidopsis transcriptome profiling indicates that multiple regulatory pathways are activated during cold acclimation in addition to the CBF cold response pathway. Plant Cell 14, 1675–1690. doi: 10.1105/tpc.003483 12172015 PMC151458

[B18] Gale M. Devos K. (1998). Plant comparative genetics after 10 years. Sci 282, 656–659. doi: 10.1126/science.282.5389.656 9784118

[B19] Gonçalves S. L. Farias J. R. B. Sibaldelli R. N. R. (2021). Soybean production and yield in the context of global climatic changes. CABI Rev. 2021, 1–10 doi: 10.1079/PAVSNNR20211601

[B20] Grant D. Nelson R. T. Cannon S. B. Shoemaker R. C. (2010). SoyBase, the USDA-ARS soybean genetics and genomics database. Nucleic Acids Res. 38, D843–D846. doi: 10.1093/nar/gkp798 20008513 PMC2808871

[B21] Habibi F. Ramezanian A. Rahemi M. Eshghi S. Guillén F. Serrano M.. (2019). Postharvest treatments with γ-aminobutyric acid, methyl jasmonate, or methyl salicylate enhance chilling tolerance of blood orange fruit at prolonged cold storage. J. Sci. Food Agric. 99, 6408–6417. doi: 10.1002/jsfa.9920 31283020

[B22] Hannah M. A. Heyer A. G. Hincha D. K. (2005). A global survey of gene regulation during cold acclimation in arabidopsis thaliana. PloS Genet. 1, e26. doi: 10.1371/journal.pgen.0010026 16121258 PMC1189076

[B23] Hu Y. Jiang L. Wang F. Yu D. (2013). Jasmonate regulates the inducer of CBF expression–c-repeat binding factor/DRE binding factor1 cascade and freezing tolerance in arabidopsis. Plant Cell 25, 2907–2924. doi: 10.1105/tpc.113.112631 23933884 PMC3784588

[B24] Ishibashi Y. Koda Y. Zheng S.-H. Yuasa T. Iwaya-Inoue M. (2013). Regulation of soybean seed germination through ethylene production in response to reactive oxygen species. Ann. Bot. 111, 95–102. doi: 10.1093/aob/mcs240 23131300 PMC3523653

[B25] Janas K. M. Cvikrová M. Pałągiewicz A. Eder J. (2000). Alterations in phenylpropanoid content in soybean roots during low temperature acclimation. Plant Physiol. Biochem. 38, 587–593. doi: 10.1016/S0981-9428(00)00778-6

[B26] Jiang H. Li C. Liu C. Zhang W. Qiu P. Li W.. (2009). Genotype analysis and QTL mapping for tolerance to low temperature in germination by introgression lines in soybean. Acta Agron. Sin. 35, 1268–1273. doi: 10.3724/SP.J.1006.2009.01268

[B27] Khalid S. Khalil T. Nasreen S. (2014). “A survey of feature selection and feature extraction techniques in machine learning,” in 2014 science and information conference: IEEE. (London, UK: IEEE). 372–378.

[B28] Kim E. Hwang S. Lee I. (2017). SoyNet: A database of co-functional networks for soybean *Glycine max* . Nucleic Acids Res. 45, D1082–D1089. doi: 10.1093/nar/gkw704 27492285 PMC5210602

[B29] Kumar S. Stecher G. Li M. Knyaz C. Tamura K. (2018). MEGA X: molecular evolutionary genetics analysis across computing platforms. Mol. Biol. Evol. 35, 1547. doi: 10.1093/molbev/msy096 29722887 PMC5967553

[B30] Lai M.-C. Lai Z.-Y. Jhan L.-H. Lai Y.-S. Kao C.-F. (2021). Prioritization and evaluation of flooding tolerance genes in soybean [*Glycine max* (L.) merr.]. Front. Genet. 11. doi: 10.3389/fgene.2020.612131 PMC7873447 33584812

[B31] Levy Y. Dodd J. Krikun J. (1983). Effect of irrigation, water salinity and rootstock on the vertical distribution of vesicular-arbuscular mycorrhiza in citrus roots. New Phytol. 95, 397–403. doi: 10.1111/j.1469-8137.1983.tb03507.x

[B32] Li J. Sun M. Liu Y. Sun X. Yin K. (2022). Genome-wide identification of wild soybean mitochondrial calcium uniporter family genes and their responses to cold and carbonate alkaline stresses. Front. Plant Sci. 1375. doi: 10.3389/fpls.2022.867503 PMC9111538 35592573

[B33] Liu X. Jin J. Wang G. Herbert S. (2008). Soybean yield physiology and development of high-yielding practices in northeast China. Field Crops Res. 105, 157–171. doi: 10.1016/j.fcr.2007.09.003

[B34] Lopes-Caitar V. S. De Carvalho M. C. Darben L. M. Kuwahara M. K. Nepomuceno A. L. Dias W. P.. (2013). Genome-wide analysis of the hsp 20 gene family in soybean: Comprehensive sequence, genomic organization and expression profile analysis under abiotic and biotic stresses. BMC Genom. 14, 1–17. doi: 10.1186/1471-2164-14-577 PMC3852298 23985061

[B35] López De Maturana E. Alonso L. Alarcón P. Martín-Antoniano I. A. Pineda S. Piorno L.. (2019). Challenges in the integration of omics and non-omics data. Genes 10, 238. doi: 10.3390/genes10030238 PMC6471713 30897838

[B36] Lukatkin A. S. Brazaityte A. Bobinas C. Duchovskis P. (2012). Chilling injury in chilling-sensitive plants: a review. Agriculture 99, 111–124.

[B37] Lyu J. Cai Z. Li Y. Suo H. Yi R. Zhang S.. (2020). The floral repressor GmFLC-like is involved in regulating flowering time mediated by low temperature in soybean. Int. J. Mol. Sci. 21, 1322. doi: 10.3390/ijms21041322 PMC7072909 32075331

[B38] Manan S. Zhao J. (2020). Role of *Glycine max* ABSCISIC ACID INSENSITIVE 3 (GmABI3) in lipid biosynthesis and stress tolerance in soybean. Funct. Plant Biol. 48, 171–179. doi: 10.1071/FP19260 32877635

[B39] Maruyama K. Todaka D. Mizoi J. Yoshida T. Kidokoro S. Matsukura S.. (2012). Identification of cis-acting promoter elements in cold-and dehydration-induced transcriptional pathways in arabidopsis, rice, and soybean. DNA Res. 19, 37–49. doi: 10.1093/dnares/dsr040 22184637 PMC3276264

[B40] Messina M. J. (1997). “Soyfoods: Their role in disease prevention and treatment,” in Soybeans (Boston: Springer), 442–477.

[B41] Ohnishi S. Funatsuki H. Kasai A. Kurauchi T. Yamaguchi N. Takeuchi T.. (2011). Variation of GmIRCHS (*Glycine max* inverted-repeat CHS pseudogene) is related to tolerance of low temperature-induced seed coat discoloration in yellow soybean. Theor. Appl. Genet. 122, 633–642. doi: 10.1007/s00122-010-1475-6 20981401

[B42] Oshunsanya S. O. Nwosu N. J. Li Y. (2019). “Abiotic stress in agricultural crops under climatic conditions,” in Sustainable agriculture, forest and environmental management. Eds. Jhariya M. Banerjee A. Meena R. Yadav D. (Singapore: Springer).

[B43] Pan W.-J. Tao J.-J. Cheng T. Shen M. Ma J.-B. Zhang W.-K.. (2017). Soybean NIMA-related kinase1 promotes plant growth and improves salt and cold tolerance. Plant Cell Physiol. 58, 1268–1278. doi: 10.1093/pcp/pcx060 28444301

[B44] Pazhamala L. T. Kudapa H. Weckwerth W. Millar A. H. Varshney R. K. (2021). Systems biology for crop improvement. Plant Genome 14, e20098. doi: 10.1002/tpg2.20098 33949787 PMC12806876

[B45] Purcell S. Neale B. Todd-Brown K. Thomas L. Ferreira M. A. Bender D.. (2007). PLINK: A tool set for whole-genome association and population-based linkage analyses. Am. J. Hum. Genet. 81, 559–575. doi: 10.1086/519795 17701901 PMC1950838

[B46] Qin P. Wang T. Luo Y. (2022). A review on plant-based proteins from soybean: Health benefits and soy product development. J. Agric. Food Res. 7, 100265. doi: 10.1016/j.jafr.2021.100265

[B47] Qiu P. Zhang W. Jiang H. Liu C. Li C. Fan D.. (2011). Genetic overlap between salt and low-temperature tolerance loci at germination stage of soybean. Sci. Agric. Sin. 44, 1980–1988.

[B48] Robison J. Arora N. Yamasaki Y. Saito M. Boone J. Blacklock B.. (2017). Cold acclimation potentials of *Glycine max* and *Glycine soja* . Crop Sci. 203, 553–561. doi: 10.1111/jac.12219

[B49] Robison J. D. Yamasaki Y. Randall S. K. (2019). The ethylene signaling pathway negatively impacts CBF/DREB-regulated cold response in soybean (*Glycine max*). Front. Plant Sci. 10. doi: 10.3389/fpls.2019.00121 PMC6396728 30853961

[B50] Sanghera G. S. Wani S. H. Hussain W. Singh N. (2011). Engineering cold stress tolerance in crop plants. Curr. Genom. 12, 30–43. doi: 10.2174/138920211794520178 PMC3129041 21886453

[B51] Schmutz J. Cannon S. B. Schlueter J. Ma J. Mitros T. Nelson W.. (2010). Genome sequence of the palaeopolyploid soybean. Nature 463, 178–183. doi: 10.1038/nature08670 20075913

[B52] Shahhosseini M. Hu G. Archontoulis S. V. (2020). Forecasting corn yield with machine learning ensembles. Front. Plant Sci. 11, 1120. doi: 10.3389/fpls.2020.01120 32849688 PMC7411227

[B53] Sharma M. Laxmi A. (2016). Jasmonates: emerging players in controlling temperature stress tolerance. Front. Plant Sci. 6. doi: 10.3389/fpls.2015.01129 PMC4701901 26779205

[B54] Smith S. E. Read D. J. (2008). Mycorrhizal symbiosis (London: Academic press).

[B55] Song L. Chen W. Wang B. Yao Q.-M. Valliyodan B. Bai M.-Y.. (2019). GmBZL3 acts as a major BR signaling regulator through crosstalk with multiple pathways in *Glycine max* . BMC Plant Biol. 19, 1–15. doi: 10.1186/s12870-019-1677-2 30795735 PMC6387493

[B56] Sun M. Jing Y. Wang X. Zhang Y. Zhang Y. Ai J.. (2020). Gma-miR1508a confers dwarfing, cold tolerance, and drought sensitivity in soybean. Mol. Breed. 40, 1–13. doi: 10.1007/s11032-020-01116-w

[B57] Tian X. Liu Y. Huang Z. Duan H. Tong J. He X.. (2015a). Comparative proteomic analysis of seedling leaves of cold-tolerant and-sensitive spring soybean cultivars. Mol. Biol. Rep. 42, 581–601. doi: 10.1007/s11033-014-3803-4 25359310

[B58] Tian X. Wang Z. Li X. Lv T. Liu H. Wang L.. (2015b). Characterization and functional analysis of pyrabactin resistance-like abscisic acid receptor family in rice. Rice 8, 1–13. doi: 10.1186/s12284-015-0061-6 PMC4567572 26362328

[B59] Vailati-Riboni M. Palombo V. Loor J. J. (2017). “What are omics sciences?,” in Periparturient diseases of dairy cows (Switzerland: Springer), 1–7.

[B60] Van Ee J. H. (2009). Soy constituents: modes of action in low-density lipoprotein management. Nutr. Rev. 67, 222–234. doi: 10.1111/j.1753-4887.2009.00192.x 19335716

[B61] Verdonck T. Baesens B. óskarsdóttir M. (2021). “Special issue on feature engineering editorial,” in Mach. Learn., 1–12. doi: 10.1007/s10994-021-06042-2

[B62] Von Bertalanffy L. (1973). “The meaning of general system theory,” in General system theory: Foundations, development, applications (New York: George Braziller Inc), 30–53.

[B63] Wang X. Chang X. Jing Y. Zhao J. Fang Q. Sun M.. (2020b). Identification and functional prediction of soybean CircRNAs involved in low-temperature responses. J. Plant Physiol. 250, 153188. doi: 10.1016/j.jplph.2020.153188 32450394

[B64] Wang Y. Ling L. Jiang Z. Tan W. Liu Z. Wu L.. (2019). Genome-wide identification and expression analysis of the 14-3-3 gene family in soybean (*Glycine max*). PeerJ 7, e79500. doi: 10.7717/peerj.7950 PMC6901008 31824753

[B65] Wang J. Song L. Gong X. Xu J. Li M. (2020a). Functions of jasmonic acid in plant regulation and response to abiotic stress. Int. J. Mol. Sci. 21, 1446. doi: 10.3390/ijms21041446 PMC7073113 32093336

[B66] Wang Q. Zhang F. Smith D. L. (1996). Application of GA3 and kinetin to improve corn and soybean seedling emergence at low temperature. Environ. Exp. Bot. 36, 377–383. doi: 10.1016/S0098-8472(96)01028-3

[B67] Wang N. Zhong X. Cong Y. Wang T. Yang S. Li Y.. (2016). Genome-wide analysis of phosphoenolpyruvate carboxylase gene family and their response to abiotic stresses in soybean. Sci. Rep. 6, 1–14. doi: 10.1038/srep38448 27924923 PMC5141416

[B68] Xia J. Zhang X. Yuan D. Chen L. Webster J. Fang A. C. (2013). Gene prioritization of resistant rice gene against xanthomas oryzae pv. oryzae by using text mining technologies. BioMed. Res. Int. 2013, 853403. doi: 10.1155/2013/853043 PMC3859262 24371834

[B69] Xu S. Liu N. Mao W. Hu Q. Wang G. Gong Y. (2016). Identification of chilling-responsive microRNAs and their targets in vegetable soybean (*Glycine max* l.). Sci. Rep. 6, 1–12. doi: 10.1038/srep26619 27216963 PMC4877674

[B70] Yamasaki Y. Koehler G. Blacklock B. J. Randall S. K. (2013). Dehydrin expression in soybean. Plant Physiol. Biochem. 70, 213–220. doi: 10.1016/j.plaphy.2013.05.013 23792826

[B71] Yamasaki Y. Randall S. K. (2016). Functionality of soybean CBF/DREB1 transcription factors. Plant Sci. 246, 80–90. doi: 10.1016/j.plantsci.2016.02.007 26993238

[B72] Yang C. Shen W. Chen H. Chu L. Xu Y. Zhou X.. (2018). Characterization and subcellular localization of histone deacetylases and their roles in response to abiotic stresses in soybean. BMC Plant Biol. 18, 1–13. doi: 10.1186/s12870-018-1454-7 30305032 PMC6180487

[B73] Yu G.-H. Jiang L.-L. Ma X.-F. Xu Z.-S. Liu M.-M. Shan S.-G.. (2014). A soybean C2H2-type zinc finger gene GmZF1 enhanced cold tolerance in transgenic arabidopsis. PloS One 9, e109399. doi: 10.1371/journal.pone.0109399 25286048 PMC4186855

[B74] Zhai J. Tang Y. Yuan H. Wang L. Shang H. Ma C. (2016). A meta-analysis based method for prioritizing candidate genes involved in a pre-specific function. Front. Plant Sci. 7. doi: 10.3389/fpls.2016.01914 PMC5156684 28018423

[B75] Zhang W. B. Qiu P. C. Jiang H. W. Liu C. Y. Xin D. W. Li C. D.. (2012). Dissection of genetic overlap of drought and low-temperature tolerance QTLs at the germination stage using backcross introgression lines in soybean. Mol. Biol. Rep. 39, 6087–6094. doi: 10.1007/s11033-011-1423-9 22207180

[B76] Zhang S. Wang Y. Li K. Zou Y. Chen L. Li X. (2014). Identification of cold-responsive miRNAs and their target genes in nitrogen-fixing nodules of soybean. Int. J. Mol. Sci. 15, 13596–13614. doi: 10.3390/ijms150813596 25100171 PMC4159813

[B77] Zhao C. Zhang Z. Xie S. Si T. Li Y. Zhu J.-K. (2016). Mutational evidence for the critical role of CBF transcription factors in cold acclimation in arabidopsis. Plant Physiol. 171, 2744–2759. doi: 10.1104/pp.16.00533 27252305 PMC4972280

