# Supplementary material for: An advanced systems biology framework of feature engineering for cold tolerance genes discovery from integrated omics and non-omics data in soybean
Source: Front Plant Sci. 2022 Sep 30;13:1019709. doi: 10.3389/fpls.2022.1019709 (PMC9562094; doi:10.3389/fpls.2022.1019709)
Supplement: Supplementary file 1 [file DataSheet_1.pdf]

Table S1. The association-based scoring system in data harmonization process

Layer	Features	Scoring method	Scoring range
DNA	SNP, QTL	$S_p = I_{p \geq 10^{-8}} \times (-\,log(p)) + I_{10^{-10} \leq p < 10^{-8}} \times 9 + I_{p < 10^{-10}} \times 10$<br>$S_{LOD} = I_{LOD \leq 8} \times \lfloor LOD \rfloor + I_{8 < LOD \leq 10} \times 9 + I_{LOD > 10} \times 10$<br>$S = \max\{S_p, S_{LOD}\}$	[0, 10]
RNA	mRNA,<br>circRNA,<br>miRNA	$S_p^{mRNA} = I_{p \geq 10^{-8}} \times (-\,log(p)) + I_{10^{-10} \leq p < 10^{-8}} \times 9 + I_{p < 10^{-10}} \times 10$<br>$S_{FC}^{mRNA} = \frac{\lvert FC \rvert}{2}$<br>$S^{mRNA} = I_{\text{FC absent}} \times \max\{S_p^{mRNA}, 1\} + I_{\text{FC present}} \times \min\{S_{FC}^{mRNA}, 1.5\}$	[0, 10]
		$S_p^{\backslash mRNA} = I_{p \geq 10^{-6}} \times (-\,log(p)) + I_{p < 10^{-6}} \times 6$<br>$S_{FC}^{\backslash mRNA} = \lfloor FC \rceil$<br>$S_{score}^{\backslash mRNA} = I_{\text{present in 2 experiments}} \times 4 + I_{\text{present in 1 experiment only}} \times 2$<br>$S^{\backslash mRNA} = \max\{S_p^{\backslash mRNA}, S_{FC}^{\backslash mRNA}, S_{score}^{\backslash mRNA}\}$	[0, 6]
Protein	Protein	$S = I_{\text{degree}<100 \text{ and } \text{CC}<0.5} \times 1 + I_{\text{degree}<100 \text{ and } \text{CC}\geq 0.5} \times 2 +$<br>$I_{\text{degree}\in\langle 100,1000) \text{ and } \text{CC}<0.1} \times 3 + I_{\text{degree}\in\langle 100,1000) \text{ and } \text{CC}\geq 0.1} \times 4 +$<br>$I_{\text{degree}\geq 1000 \text{ and } \text{CC}<0.01} + I_{\text{degree}\geq 1000 \text{ and } \text{CC}\geq 0.01}$	[0, 6]
Function	Metabolites	$S = I_{p \geq 10^{-6}} \times (-\,log(p)) + I_{p < 10^{-6}} \times 6$	[0, 6]
Homologs	Gene	$S = I_{1 \leq \text{ref} \leq 3} \times 1 + I_{3 < \text{ref} \leq 6} \times 2 + I_{6 < \text{ref} \leq 10} \times 3 + I_{10 < \text{ref} \leq 20} \times 4 + I_{20 < \text{ref} \leq 100} \times 5 + I_{100 < \text{ref}} \times 6$	[0, 6]

Abbreviation: SNP, single nucleotide polymorphism; QTL, quantitative trait locus; mRNA, messenger RNA; circRNA, circular RNA; miRNA, micro RNA; *p*, *p*-value; LOD, logarithm of odds; FC, fold change; \mRNA, data excluding mRNA feature in RNA layer; CC, cluster coefficient; ref, reference number.

Table S2. Functional pathways related to cold tolerance or response to cold stress

**Functional pathway**[a]	**CTgenes involved in the pathway**
Response to cold	*Glyma.02g005600*, *Glyma.03g250600*, *Glyma.04g044900*, *Glyma.06g007500*, *Glyma.06g045400*, *Glyma.06g108900*, *Glyma.09g071600*, *Glyma.10g006600*, *Glyma.10g067000*, *Glyma.10g165800*, *Glyma.10g180800*, *Glyma.11g036400*, *Glyma.12g221500*, *Glyma.13g279900*, *Glyma.14g088300*, *Glyma.15g048600*, *Glyma.15g179600*, *Glyma.17g236200*, *Glyma.18g225400*, *Glyma.20g133200*, *Glyma.20g209700*
Cold acclimation	*Glyma.06g108900*, *Glyma.10g067000*, *Glyma.15g048600*
Immune-related	*Glyma.04g061400*, *Glyma.06g108900*, *Glyma.08g218600*, *Glyma.13g370100*, *Glyma.14g103100*, *Glyma.15g003300*, *Glyma.15g048600*
Response to freezing	*Glyma.01g216000*, *Glyma.05g049900*
Response to osmotic	*Glyma.06g007500*, *Glyma.06g108900*, *Glyma.15g048600*
Response to stress	*Glyma.04g044900*, *Glyma.06g045400*, *Glyma.14g088300*, *Glyma.17g236200*, *Glyma.20g133200*
Response to temperature stimulus	*Glyma.18g225400*
Defense response-related	*Glyma.01g204400*, *Glyma.02g005600*, *Glyma.03g250600*, *Glyma.04g044900*, *Glyma.04g061400*, *Glyma.06g045400*, *Glyma.06g108900*, *Glyma.08g218600*, *Glyma.09g071600*, *Glyma.10g006600*, *Glyma.10g165800*, *Glyma.10g180800*, *Glyma.11g036400*, *Glyma.12g221500*, *Glyma.13g239000*, *Glyma.13g279900*, *Glyma.13g370100*, *Glyma.14g088300*, *Glyma.14g103100*, *Glyma.15g003300*, *Glyma.15g048600*, *Glyma.15g179600*, *Glyma.17g236200*, *Glyma.18g225400*, *Glyma.20g070000*, *Glyma.20g133200*, *Glyma.20g209700*

[a]Forty CTgenes, selected from 17 enriched GO pathways, were analyzed using ‘Gene Model Data Mining and Analysis’ tool in the SoyBase. These genes were involved in 8 functional pathways.

Table S3. Enriched pathways of the CTgenes for short-, mid- and long-term cold tolerance.

**GO pathway**	N^{GO}	**Short-term**		**Mid-term**		**Long-term**	
		N^{CT}	$p^{adjusted}$	N^{CT}	$p^{adjusted}$	N^{CT}	$p^{adjusted}$
Root system development	19			10	1.20×10^{-9}		
Root meristem growth	18			10	5.83×10^{-10}		
Response to wounding	1,031			95	$<1.00\times10^{-16}$		
Response to L-glutamate	10			10	1.59×10^{-14}		
Response to jasmonic acid stimulus	756			94	$<1.00\times10^{-16}$		
Response to fungus	310			73	$<1.00\times10^{-16}$		
**Regulation of gibberellin biosynthetic process**	5	2	1.60×10^{-2}			2	2.00×10^{-2}
**Positive regulation of transcription, DNA-dependent**	1,080	15	5.30×10^{-3}			15	2.20×10^{-2}
Jasmonic acid mediated signaling pathway	800			55	1.55×10^{-9}		
Jasmonic acid biosynthetic process	417			112	$<1.00\times10^{-16}$		
**Cold acclimation**	50	6	6.81×10^{-6}			6	1.35×10^{-5}
Abscisic acid mediated signaling pathway	634			52	7.11×10^{-12}		

Abbreviation: GO, gene ontology; N^{GO}, number of genes in a particular GO terms; N^{CT}, number of CTgenes in a particular GO terms.

All *p*-values were adjusted by Bonferroni correction to account for false positive results.

Table S4. Enriched pathways of the difference sets of the top genes between the SFAF prioritization and the NPRF prioritization.

Difference set	Enriched Pathway	$\mathbf{N}^{GO}$	$\mathbf{N}^{CT}$	***p*-value$^{\mathbf{adjusted}}$**
Short-term CTgenes$^{NPRF\backslash SFAF}$	Cold acclimation	50	6	2.88×10^{-6}
	Regulation of GA biosynthesis process	5	2	4.80×10^{-3}
Mid-term CTgenes$^{NPRF\backslash SFAF}$	Response to L-glutamate	10	9	7.78×10^{-16}
	Root meristem growth	18	9	3.56×10^{-12}
	Root system development	19	9	6.64×10^{-12}
	Callose deposition in cell wall	74	9	4.91×10^{-6}
	Microsporogenesis	121	9	3.30×10^{-4}
	Jasmonic acid biosynthetic process	417	15	2.05×10^{-3}
	Cell communication	186	9	1.00×10^{-2}
	Response to fungus	310	11	2.80×10^{-2}
Long-term CTgenes$^{NPRF\backslash SFAF}$	Cold acclimation	50	6	8.16×10^{-7}
Mid-term CTgenes$^{SFAF\backslash NPRF}$	Jasmonic acid biosynthesis process	417	16	1.29×10^{-7}
	Oxidoreduction coenzyme metabolic process	121	8	3.70×10^{-5}
	Vitamin metabolic process	121	8	3.70×10^{-5}
	Lipoate metabolic process	121	8	3.70×10^{-5}
	Coenzyme biosynthetic process	121	8	3.70×10^{-5}
	Sulfur compound biosynthetic process	121	8	3.70×10^{-5}
	Aromatic amino acid family metabolic process	128	8	5.70×10^{-5}
	Secondary metabolic process	135	8	8.55×10^{-5}
	Sulfur amino acid metabolic process	137	8	9.56×10^{-5}

	Nucleotide metabolic process	150	8	1.89×10^{-4}
	Glycine catabolic process	157	8	2.70×10^{-4}
	Regulation of lipid metabolic process	81	6	6.10×10^{-4}
	Chlorophyll metabolic process	90	6	1.10×10^{-3}
	Cellular amino acid biosynthetic process	193	8	1.20×10^{-3}
	Oxylipin biosynthetic process	96	6	1.60×10^{-3}
	Unsaturated fatty acid biosynthetic process	204	8	1.80×10^{-3}
	Chlorophyll biosynthetic process	294	8	2.30×10^{-2}
	Abscisic acid biosynthetic process	53	4	2.30×10^{-2}

Abbreviation: GO, gene ontology; NPRF, non-parameter random forest; SFAF, step-function adjusted factor; N^{GO}, number of genes in a particular GO terms; N^{CT}, number of CTgenes in a particular GO terms.

$CTgenes^{NPRF\backslash SFAF}$ represents the difference set of the prioritized genes identified by the SFAF prioritization from the prioritized CTgenes identified by the NPRF prioritization. $CTgenes^{SFAF\backslash NPRF}$ represents the difference set of the prioritized CTgenes identified by the NPRF prioritization from the prioritized genes identified by the SFAF prioritization. All *p*-values were adjusted by Bonferroni correction to account for false positive results.
